# Supplementary material for: Understanding usual care for patients with multimorbidity: baseline data from a cluster-randomised trial of the 3D intervention in primary care
Source: BMJ Open. 2018 Aug 29;8(8):e019845. doi: 10.1136/bmjopen-2017-019845 (PMC6119425; doi:10.1136/bmjopen-2017-019845)
Supplement: Supplementary file 1 [file bmjopen-2017-019845supp001.pdf]

Appendix A Chronic conditions for inclusion

We collected data on diagnoses of 17 conditions and combined these into 11 groups as shown below. Included patients must have diagnoses from three or more these groups of chronic conditions:

- Cardiovascular disease or Chronic kidney disease (including coronary heart disease, hypertension, heart failure, peripheral arterial disease, chronic kidney disease stage 3 to 5)*
- Stroke
- Diabetes
- Chronic Obstructive Pulmonary Disease or Asthma*
- Epilepsy
- Atrial fibrillation
- Severe mental health problems (schizophrenia or psychotic illness)*
- Depression
- Dementia
- Learning disability
- Rheumatoid arthritis

*Groups are counted only once even if a patient has multiple conditions within a group. For example, having both hypertension and heart failure would just count for one condition
