# Supplementary material for: Understanding usual care for patients with multimorbidity: baseline data from a cluster-randomised trial of the 3D intervention in primary care
Source: BMJ Open. 2018 Aug 29;8(8):e019845. doi: 10.1136/bmjopen-2017-019845 (PMC6119425; doi:10.1136/bmjopen-2017-019845)
Supplement: Supplementary file 2 [file bmjopen-2017-019845supp002.pdf]

Appendix B - 3D Study questionnaire

Practice: **Date:**

Please answer this questionnaire thinking about the patients with multi-morbidity in your practice. This will help us to understand your current approach and your perceptions about the care of these patients.

Name (optional): **Role (required):**

Please place an X in the box that is closest to your opinion	Strongly disagree	Disagree	Neither agree or disagree	Agree	Strongly agree
1. Patients' main concerns may be overlooked during review of their long-term conditions					
2. Depression is difficult to identify reliably without using a measure (such as PHQ9)					
3. Poly-pharmacy is difficult for patients to manage					
4. Multi-morbidity is difficult for clinicians to manage					
5. Patients with multi-morbidity have a special need for holistic, patient-centred care					
6. Holistic, patient-centred care is enhanced by continuity of care					
7. Patients with 3 or more conditions need longer appointments to address all their concerns					
8. Patients being reviewed for a long-term condition should be given a written care plan					
9. Patients prefer it if I make a plan, instead of asking them what they would like to do					
10. Patients are more likely to keep to goals and plans that they suggest themselves					
11. In this practice, the care patients receive for their long-term conditions is well-co-ordinated					
12. In this practice, review of long-term conditions is too disease-orientated and not holistic enough.					

Thank you for completing this questionnaire! Please return it to the 3D research team.
