# Supplementary material for: Understanding usual care for patients with multimorbidity: baseline data from a cluster-randomised trial of the 3D intervention in primary care
Source: BMJ Open. 2018 Aug 29;8(8):e019845. doi: 10.1136/bmjopen-2017-019845 (PMC6119425; doi:10.1136/bmjopen-2017-019845)
Supplement: Author's manuscript [file bmjopen-2017-019845.draft_revisions.pdf]

BMJ Open

BMJ Open is committed to open peer review. As part of this commitment we make the peer review history of every article we publish publicly available.

When an article is published we post the peer reviewers' comments and the authors' responses online. We also post the versions of the paper that were used during peer review. These are the versions that the peer review comments apply to.

The versions of the paper that follow are the versions that were submitted during the peer review process. They are not the versions of record or the final published versions. They should not be cited or distributed as the published version of this manuscript.

BMJ Open is an open access journal and the full, final, typeset and author-corrected version of record of the manuscript is available on our site with no access controls, subscription charges or pay-per-view fees (http://bmjopen.bmj.com).

If you have any questions on BMJ Open's open peer review process please email info.bmjopen@bmj.com

BMJ Open

Understanding usual care for patients with multimorbidity: baseline data from a cluster randomised trial of the 3D intervention in primary care

Journal:	*BMJ Open*
Manuscript ID	bmjopen-2017-019845
Article Type:	Research
Date Submitted by the Author:	28-Sep-2017

Complete List of Authors:	Chaplin, Katherine ; University of Bristol Centre for Academic Primary Care, Population Health Sciences, Bristol Medical School,<br>Bower, Peter; University of Manchester, NIHR School for Primary Care Research, Centre for Primary Care, Division of Population of Health, Health Services Research and Primary Care, Manchester Academic Health Science Centre<br>Man, Mei-See; University of Bristol Centre for Academic Primary Care, Population Health Sciences, Bristol Medical School, ; University of Bristol, Bristol Randomised Trials Collaboration (BRTC), Population Health Sciences, Bristol Medical School<br>Brookes, Sara ; University of Bristol, Bristol Randomised Trials Collaboration (BRTC), Population Health Sciences, Bristol Medical School<br>Gaunt, Daisy; University of Bristol, Bristol Randomised Trials Collaboration (BRTC), Population Health Sciences, Bristol Medical School<br>Guthrie, Bruce; University of Dundee, Population Health Sciences Division, School of Medicine,<br>Mann, Cindy; University of Bristol Centre for Academic Primary Care, Population Health Sciences, Bristol Medical School,<br>Mercer, Stewart; University of Glasgow, Institute of Health and Wellbeing<br>Rafi, Imran; Royal College of General Practitioners, Clinical Innovation and Research<br>Shaw, Ali; University of Bristol Centre for Academic Primary Care, Population Health Sciences, Bristol Medical School,<br>Salisbury, Chris; University of Bristol Centre for Academic Primary Care, Population Health Sciences, Bristol Medical School,

Keywords:	Chronic disease, Family practice, Multimorbidity, Patient centred care, Comorbidity

SCHOLARONE™
Manuscripts

TITLE: Understanding usual care for patients with multimorbidity: baseline data from a cluster randomised trial of the 3D intervention in primary care

Katherine Chaplin[1], Pete Bower[2], Mei-See Man[1&3], Sara Brookes[3], Daisy Gaunt[3], Bruce Guthrie[4], Cindy Mann[1], Stewart Mercer[5], Imran Rafi[6], Alison Shaw[1], Chris Salisbury[1]

Corresponding author:

Prof Chris Salisbury,

Centre for Academic Primary Care, Population Health Sciences, Bristol Medical School

Canynge Hall, 39 Whatley Road,

University of Bristol, Bristol, UK

c.salisbury@bristol.ac.uk

Tel 0117 3314543

Co-authors:

[1] Centre for Academic Primary Care, Population Health Sciences, Bristol Medical School, University of Bristol, Bristol, UK

[2] NIHR School for Primary Care Research, Centre for Primary Care, Division of Population of Health, Health Services Research and Primary Care, Manchester Academic Health Science Centre, University of Manchester, Manchester, UK

[3] Bristol Randomised Trials Collaboration (BRTC), Population Health Sciences, Bristol Medical School, University of Bristol, Bristol, UK

[4] Population Health Sciences Division, School of Medicine, University of Dundee, Dundee, UK

[5] Institute of Health and Wellbeing, General Practice and Primary Care, University of Glasgow, 1 Horslethill Road, Glasgow G12 9LX

[6] Clinical Innovation and Research, Royal College of General Practitioners, Euston Square, London, NW1 2FB

Keywords (up to 5): Chronic disease. Family practice. Multimorbidity. Patient centred care. Comorbidity

Word count (excl title page, abstract & references) 3606

Number of figures = 2

Number of tables/boxes = 6

For peer review only

Number of supplementary files = 2

Number of references = 37

For peer review only

Abstract

Objectives

Recent evidence has highlighted the high prevalence and impact of multimorbidity, but the evidence base for improving management is limited. We have tested a new complex intervention for multimorbidity (the 3D model). The paper describes the baseline characteristics of practices and patients which participated in the trial. It also explores current 'usual primary care' for multimorbidity, against which the 3D intervention is being tested.

Design

Study using baseline data from patients in a trial and additional data from practice staff

Setting

Primary care in the United Kingdom

Participants

Patients with multimorbidity, and practice staff in primary care

Primary and secondary outcome measures

Using surveys and routinely available data, we compared the characteristics of participating and non-participating practices and compared the characteristics of patients at each stage of recruitment to the trial.

Using baseline questionnaire data from the trial we present patient-reported data about participant illness burden, treatment burden and perceptions of receiving patient-centred care. We obtained data about usual care from practice staff using questionnaires and a structured proforma.

Results

Participating practices were slightly larger, in less deprived areas, and with slightly higher scores for patient satisfaction compared with non-participating practices. Comparison of participants with non-participants identified only minor differences in characteristics, suggesting that the sample was representative. Patients reported significant levels of illness burden, and an important minority reported treatment burden in relation to issues such as medication. Although patients reported relatively high levels of satisfaction with care, many patients reported not having received potentially important components of care for multimorbidity.

Conclusion

The data suggest our trial achieved good levels of external validity. Although patients were generally very satisfied with primary care services, the data suggest that there was significant room for improvement in important aspects of care for multimorbidity that are targeted by the 3D intervention.

[299/300 words]

For peer review only

Strengths and limitations of this study

- Data on the external validity of trial populations is often not available, but recruitment using routine GP records allowed us to compare participants and non-participants
- We collected detailed data on care for multimorbidity using validated scales, complemented with data from staff for a more comprehensive assessment.
- Comparisons of participants and non-participants were limited to data available in routine records
- Data on delivery and quality of care were generally based on patient and clinician self-report.

For peer review only

Introduction

Recent evidence has highlighted the importance of multimorbidity for current health policy.[1] Multimorbidity among long-term conditions is the norm among older patients, and is common at a younger age in deprived populations.[2 3] It is associated with significant impacts on quality of life, mortality, and health care utilisation. [1]

There is increasing consensus on the sort of care that is required for the management of patients with multimorbidity.[4] Much of this derives from consensus about high quality care for long-term conditions more generally, with a focus on care planning, shared decision-making, and self-management. However, management of patients with multimorbidity also raises specific challenges, such as how to prioritise among conditions, and how best to manage the treatment burden experienced due to multiple treatments and multiple appointments. The increased prevalence of depression in multimorbidity is well recognised, and comorbid depression is associated with worse outcomes.[5]

However, the evidence base for the management of multimorbidity remains sparse. A recent Cochrane review reported only 18 randomised trials specifically targeting multimorbidity, and concluded that 'there are remaining uncertainties about the effectiveness of interventions for people with multimorbidity in general due to the relatively small number of RCTs conducted in this area to date.[6 7] The National Institute for Health and Care Excellence (NICE - the leading UK organisation for the development of clinical guidelines) has published guidelines for the clinical assessment and management of multimorbidity, reviewing the evidence for varying 'format of encounters' in people with multimorbidity (including longer consultations, structured recall, interventions to involve the patient in agenda-setting, and multi-professional appointments) and for primary care based comprehensive geriatric assessment.[1] However, the evidence available did not support any specific recommendation on how to organise primary care to better meet the needs of people with multimorbidity. Instead the guideline development group made a recommendation for research into alternative approaches to organising primary care compared to usual care for people with multimorbidity.

The Cochrane review suggested that, given the complexity of needs and management of patients with multimorbidity, interventions are likely to be 'complex' (i.e. 'involving several components acting in concert to improve care').[6 7] Our team has developed the 3D model for the management of multimorbidity in primary care. The model is described in full elsewhere,[8] and is currently undergoing evaluation in a large scale randomised controlled trial (ISRCTN06180958).[8]

The problems posed by current healthcare organisation and experienced by patients with multimorbidity can be summarised as a lack of holistic patient-centred care, a high burden of illness and a high level of treatment burden due to multiple medications and the need to attend numerous appointments. Figure 1 shows how the 3D approach addresses these problems. The basis for 3D is the patient-centred care model, [9-11] which includes four components:

- A focus on the patient's individual disease and illness experience
- A biopsychosocial perspective
- Finding common ground on what the problem is and mutually agreeing management plans
- Enhancing the relationship between the patient and doctor (the therapeutic alliance)

<Insert Figure 1>

For peer review only

The Medical Research Council has a well-developed framework for the development, evaluation and description of complex interventions.[12] Recent work in this area has also emphasised two additional issues. First, there is a need to understand the practice and patient populations who actually enter trials of complex interventions, compared to those who are potentially eligible, to better understand the external validity of the study.[13 14] Secondly, there is a need to better understand the comparator to the intervention (in this case, 'usual primary care') in order to understand the content and quality of care against which the complex intervention is being tested.[15] The aims of this study are therefore to:

1. Compare practices and patients participating in the trial with non-participants

2. Describe the characteristics of participating patients at baseline in terms of their experiences of (a) illness burden (b) treatment burden and (c) patient centred care

3. Describe usual care for people with multimorbidity

For peer review only

Methods

Design

The design of the 3D trial has been described in full[8] and is briefly summarised here. The 3D trial is a multi-centre pragmatic, two-arm, practice-level cluster randomised controlled trial. This study is based in general practices in three areas; Bristol; Greater Manchester; and Ayrshire and Arran. Volunteer practices were recruited from areas with a range of socioeconomic characteristics, with no inclusion or exclusion criteria except the use of the EMIS clinical IT system (used by the majority of practices). Inclusion criteria for patients were age 18+ and having three or more long-term conditions from those included in the NHS Quality and Outcomes Framework (QOF) (Appendix A). Up to 150 potentially eligible patients were randomly selected from each practice and screened by their GPs. Exclusion criteria were: having a life expectancy of less than 12 months; serious suicidal risk; known to be leaving the practice within 12 months; unable to complete questionnaires in English even with the help of carers; actively taking part in other research involving extra visits to primary care or other health services; lacking capacity to consent (Scotland only); or being considered unsuitable for the research study by their GP. All remaining patients were sent an invitation including information about the study, a consent form and baseline questionnaire. Non-respondents were sent one postal reminder 10-14 days later, supplemented by a telephone reminder in those practices where recruitment targets are not met.

Patient data

We had data on two groups of patients. For patients who were invited to the trial ('potentially eligible patients'), we had data on age, sex and conditions.

For eligible patients who consented to take part ('participating patients'), details of medical conditions were collected from medical records. Additionally participating patients completed a baseline questionnaire measuring depression (Hospital Anxiety and Depression Scale)[16], quality of life (EQ5D-5L)[17], illness burden (Bayliss)[18], treatment burden (MTBQ) (submitted for publication)), patients' perception of the quality of chronic illness care (PACIC)[19] and perceived empathy of GPs and nurses (CARE).[20]

The questionnaire included several questions about holistic patient centred care. These included questions within the PACIC measure and the CARE measure, along with two questions from LTC6 Quality Innovation Productivity and Prevention (QIPP) programme. Three further questions were included regarding satisfaction with current care, whether patients usually saw their preferred GP, and whether they had a written care plan.

Staff perceptions and practice data on the organisation of care

At the start of the trial, participating GPs and practice nurses completed a purpose-designed questionnaire about their beliefs and attitudes regarding care of patients with multimorbidity. Researchers training the nurses and GPs in intervention practices asked them to complete the questionnaire before the training began. In usual care practices the questionnaire was distributed via the practice manager and where there was a poor response the researcher followed up the request with one reminder. The questionnaire consisted of 12 statements that were scored from 1 ('strongly disagree') to 5 ('strongly agree').

In addition, information about how the practice organised usual care for patients with long-term conditions was collected from all practices through a structured proforma completed by a single key

respondent in each practice (usually the practice manager). This covered staff resources, organisation of long term condition review clinics and practice policy on medication reviews, care plans and continuity of care. Data were collected through an emailed survey supplemented by telephone or face-to-face interview to obtain further details.

Analysis

In order to compare practices and patients participating in the trial with non-participants we compared the characteristics of practices in the 3D trial with practices in the same Clinical Commissioning Group (CCG) and national data. We assessed differences in patient populations (age, deprivation), practice characteristics (size, patient satisfaction) and published assessments of quality (QOF achievement).[21]

We described the demographic and clinical characteristics of patients at each stage of recruitment to 3D – those identified as potentially eligible but excluded by their GP, those eligible but not participating (due to non-response or actively declining), and those who agreed to participate in the study. For participants in the trial we present descriptive data on patients self-reported baseline measures of their illness burden and treatment burden.

To describe the extent to which current care for people with multimorbidity is patient-centred from the perspective of patients we present participant responses to individual question items from the baseline patient questionnaire reflecting key concepts in patient centred care.

We also provide data about staff views about care for people with multimorbidity and report descriptive data from the structured proforma about usual care for patients with multimorbidity in all practices participating in the trial.

For peer review only

Results

What types of practices and patients participated in the 3D trial, and how did they compare to non-participants?

Across the 3 sites, 35 practices signed up to the study. Two practices subsequently withdrew prior to randomisation. The remaining 33 practices (24% of those approached) were randomised, 16 into the intervention arm and 17 to usual care. Descriptive characteristics of the 33 practices are shown in Table 1. Compared with all practices in their local area, practices which agreed to participate tended to be slightly larger, in less deprived areas, and had slightly higher scores for patient satisfaction (Table 1).

<Insert Table 1>

The flow of patients into the trial is shown in Figure 2. A total of 9772 patients were identified as potentially eligible, representing 3.9% of the adult population. Of these, 5253 were randomly sampled from practice registers. GPs excluded 575 (11%) of those based on medical record data because they were ineligible or the GP felt it would be inappropriate to invite them to participate. Potential participants who were excluded by their GPs were much more likely to have dementia or learning difficulties and less likely to have diabetes or respiratory conditions than those not excluded (Table 2).

<Insert Figure 2>

Of 4678 patients invited to participate, 1546 (33%) provided consent. Patients who participated had similar health conditions to non-participants, except that participants were slightly less likely to have depression, severe mental health conditions or learning difficulties. Of the 11 types of long-term condition which made people eligible for the trial the most commonly reported were cardiovascular disease (including hypertension, peripheral artery disease, chronic kidney disease, coronary heart disease and heart failure; affecting 93% of participants), diabetes (52%) and respiratory conditions (asthma or chronic obstructive pulmonary disease; 50%).

Baseline demographic and health data on excluded patients, non-participants and participants are shown in Table 2. Excluded patients were more likely to be female, older and have 4 or more conditions than those invited. Participants and non-participants had very similar characteristics, except that the participants were slightly more likely to be male.

<Insert Table 2>

Baseline characteristics of participating patients in terms of (a) illness burden and (b) treatment burden

Two thirds of patients (66%) reported having fair or poor health, with less than 7% reported having very good or excellent health (Table 3). Although inclusion to the trial was based on QOF conditions in medical records, patients self-reported an average of seven conditions from the more comprehensive list included in the Bayliss measure.[18] Based on the HADs measure, more than a third of patients (38%) reported anxiety or depression of at least mild severity.

<Insert Table 3>

For peer review only

On average patients reported regularly taking eight medications with 32% of patients taking at least 10 regular medications (Table 4). More than half (55%) reported at least a moderate level of treatment burden, with a score of at least ten on the MTBQ. This score would be achieved, for instance, by having some difficulty in at least two areas of health care, or severe difficulty in at least one area.

<Insert Table 4>

The extent to which current care for people with multimorbidity is patient-centred from the perspective of patients

Table 4 shows that most patients indicated that a GP or primary care nurse was responsible for their long-term condition, and reported relatively high levels of overall satisfaction with their care, although reported levels of care co-ordination were somewhat lower. Three quarters had a preferred GP and of these 66% saw that GP 'most of the time'. In terms of 'whole person care', approximately two thirds of patients reported that their GP and nurse were 'excellent' or 'very good' at 'being interested in them as a whole person'. However, only 37% reported that their care was always 'joined up'.

The data show that many patients do not perceive care as patient centred in terms of focusing on an individual's experience, finding common ground and agreeing management plans. A relatively high proportion of patients (35%) reported 'rarely' or 'not at all' discussing what was most important to them in terms of their health (Table 4). Only 10% of participants reported having a care plan. Scores on the PACIC scale were around the mid-point of the scale, with the highest ratings for 'activation' and 'decision support', and the lowest for 'goal setting' and 'follow up' (Table 4).

The extent to which current care for people with multimorbidity is patient-centred from the perspective of primary care clinicians

The vast majority (88%) of clinicians agreed that patients with multimorbidity have a special need for patient centred care and over 95% agreed that continuity of care improves patient-centred care (Table 5). Most clinicians agreed that patients with a long-term condition should be given a care plan and that they were more likely to adhere to goals they had suggested themselves, but were evenly divided on whether patients preferred the clinician to make the plan. More than half of the clinicians agreed that patients' main concerns may be overlooked in long-term condition reviews (Table 5). Almost all clinicians (93%) felt that patients with multimorbidity need longer appointments to address all of their concerns.

<Insert Table 5>

The extent to which current usual care aligns with the 3D model, on the basis of practice policies

Only one of the 33 practices said they routinely provided patients with a written care plan (and 80% of the patients in that practice said they did not have a written care plan). Only one third (n=10) of practices had an active policy to encourage continuity of care, with the majority of others saying they try to accommodate patient preference. Only 36% of practices said they routinely performed depression screening while 76% said they conducted face to face medication reviews at least annually. All except two practices said they tried to combine reviews of some long-term conditions which might lessen treatment burden and improve joined up care.

<Insert Table 6>

For peer review only

Discussion

Summary of the findings

The paper describes usual care for people with high-levels of multimorbidity using baseline data from a cohort of patients entering a trial. Comparison of patients entering the trial with non-participants identified only minor differences in demographic and clinical characteristics, suggesting that external validity for this trial would be high. As anticipated, participants in the trial reported high levels of illness burden and treatment burden. Although participants reported relatively high levels of satisfaction with their relationships with professionals, the more detailed responses to specific questions identified important gaps in the extent to which they experience care as patient-centred. Although clinicians supported aspects of patient-centred care such as continuity of care and care plans, and claimed to provide these, the experiences of patients were variable. The results of this study suggest that there is significant room for improvement in many aspects of care for multimorbidity that are targeted by the 3D intervention.

Strengths and limitations

A key strength was our ability to collect comparative data on 'potentially eligible' patients, to allow us to compare participants and non-participants. Data on the external validity of trial populations is often not available, but recruitment using routine GP records does provide significant advantages in this regard. We also collected detailed data on care for multimorbidity using validated scales, and complemented these with data from staff to provide a more comprehensive assessment.

Detailed comparisons of participants and non-participants are inevitably difficult because more detailed survey data are by definition not available for non-participants, and comparisons are restricted to basic demographic characteristics. However in this study we have used anonymised practice records to compare clinical diagnoses and been able to show that participants have similar characteristics to non-participants. The bulk of the findings in this study about patient centred aspects of care come from self-report from patients and professionals, and we do not know how these relate to actual delivery of care in these practices. However, a key aim of the intervention is to improve patient experience of care, so self-report is the optimal method for assessing that.

As a pragmatic trial, 3D is designed to recruit a population with high external validity by ensuring that practices and patients who participate are representative of the wider population to whom the intervention, if effective, would be provided in real life. The overall response rate among patients invited was 33%. This is likely to be an under-estimate of the proportion of eligible patients recruited because some non-responders may not have been eligible. Nevertheless, this recruitment rate is typical of previous studies in UK populations of primary care patients with long-term conditions,[22 23] and may be considered relatively high given that the inclusion criteria for this trial selected elderly patients with multiple illnesses.

Interpretation of the findings and comparisons with the wider literature

We raised three main issues in this paper. First, how do practices and patients in 3D compare to the wider primary care population outside the trial? Although limited by available data, the comparisons suggested that the consenting sample did not differ markedly from the potentially eligible population on measured characteristics, with the largest difference being the proportion with dementia, which is unsurprising given the nature of the recruitment method. Although we cannot be sure that patients agreeing to take part do not differ on other important characteristics, the data do

provide some confidence that the results are not based on a highly selected sample, especially in terms of physical health conditions.

The second issue is the levels of illness burden, treatment burden and patient-centred care experienced by patients with multimorbidity. Our recruitment method used a simple method of condition counts which is easy to conduct, but it was unclear whether we would identify patients with high needs. In terms of illness burden, our data suggest a sample with relatively high level of morbidity and need. Patients report an average of seven conditions, and nearly two thirds report general health that is either 'fair' or 'poor'. Patients were receiving a large number of medications and more than a third of participants reported anxiety or depression. Examining the baseline data also demonstrates that, consistent with previous literature, patients with multimorbidity are burdened by the demands placed on them by treatment and expectations of self-management.[24 25] Although there are many qualitative papers on the experience of patients with multimorbidity,[26] more quantitative data is needed. The trial recruitment procedures therefore identified a group of patients with significant burdens of illness and treatment whose characteristics seem well matched to the intervention model, and where many patients exceed minimum requirements of the trial eligibility criteria. Our data also suggest that patients do not receive care which they perceive as patient-centred in several important respects, as discussed below.

The third issue raised by this paper is an understanding of 'usual primary care' for multimorbidity in this population, to better understand current practice against which the potential benefits of 3D are being assessed. Assessing the 'nature of current care' for multimorbidity, and the degree to which it is 'patient centred care' is a complex task. Nevertheless, several important findings can be highlighted, linked to the 3D model (Figure 1).

Most patients reported satisfaction in general with their care. These high ratings are in line with wider work on patient perceptions of primary care and might indicate limited scope for improvement, but interpretation of such satisfaction scores is not always straightforward to interpret.[27] However, when considering the more structured aspects of care for long-term conditions (as assessed in models such as the Chronic Care Model[28]), the results showed more room for improvement. Many patients reported that their care was not always joined up and although three quarters of patients in this study had a preferred GP, only 59% reported that they usually consult them. The 3D model identifies eliciting and responding to the patient agenda (their own individual priorities) as a key gap in current care, and the questions from the LTC6 questionnaire and the PACIC scale showed only modest levels of agreement about items relating to this facet of care. This is in line with previous work in a broader population of patients.[29] Similarly, despite a very significant policy focus on care plans,[30] many practices did not have a policy to provide them and most patients did not report receiving them.

Many of the processes of care where we identified gaps (such as improving continuity and co-ordination of care, establishing the patient agenda to improve shared decision-making, production of care plans,) are a focus of the 3D model. If these processes are mediators of improvements in quality of life, as hypothesised by the logic model underlying the 3D approach, the trial may have a reasonable chance of seeing change in the intended primary and secondary outcomes, assuming it can be implemented.

Summary

The data suggest our pragmatic trial has achieved reasonable levels of external validity, and that the results should be generalizable to primary care in the United Kingdom. Although patients were generally satisfied with their relationships with primary care professionals, there remains significant

room for improvement in important aspects of care for multimorbidity that are targeted by the 3D intervention. The pragmatic 3D randomised controlled trial will both test whether our intervention can generate enhancements in those processes of care, and whether those enhancements translate to better patient quality of life, patient experience and value for money.

Acknowledgements

The authors would like to thank Bristol Clinical Commissioning Group (CCG) for hosting this research, in particular Emma Moody, Joanne Atkinson, and Rebecca Robinson. We thank PRIMIS for developing the search. We would also like to thank members of the independent TSC, DMC, advisory group, and public and patient involvement group for their advice and input into the design and conduct of the study. Finally we would like to thank the practices and patients and trainers for their participation.

Funding statement

This project was funded by the National Institute for Health Research Health Services and Delivery Research Programme (project number 12/130/15).

Contributors

CS conceived the original study. CS, PB, SM, BG, IR, SB, AS and CM are co-applicants on the funding application. KC along with PB and CS led the writing of the first draft of the paper with contribution from DG (statistical analyses). All authors contributed to the development of the editing of this manuscript.

Disclaimer

The views and opinions expressed therein are those of the authors and do not necessarily reflect those of the HS&DR Programme, NIHR, NHS or the Department of Health.

Competing interests None declared.

Ethics approval South-West (Frenchay) NHS Research Ethics Committee (14/SW/0011) and local NHS R&D approvals from the appropriate participating trusts. The views and opinions expressed therein are those of the authors and do not necessarily reflect those of the HS&DR Programme, NIHR, NHS or the Department of Health.

Sponsor

The trial sponsor is the University of Bristol, (Senate House, Tyndall Avenue, Bristol BS8 1TH, UK)

Provenance and peer review Not commissioned; externally peer reviewed.

Data sharing statement Once the main results have been published, data may be available to other investigators subject to agreement about the protocol with the chief investigator and compliance with policies of the funder and sponsor in relation to data sharing.

Figure 1: 3D logic model including theoretical mechanisms of action

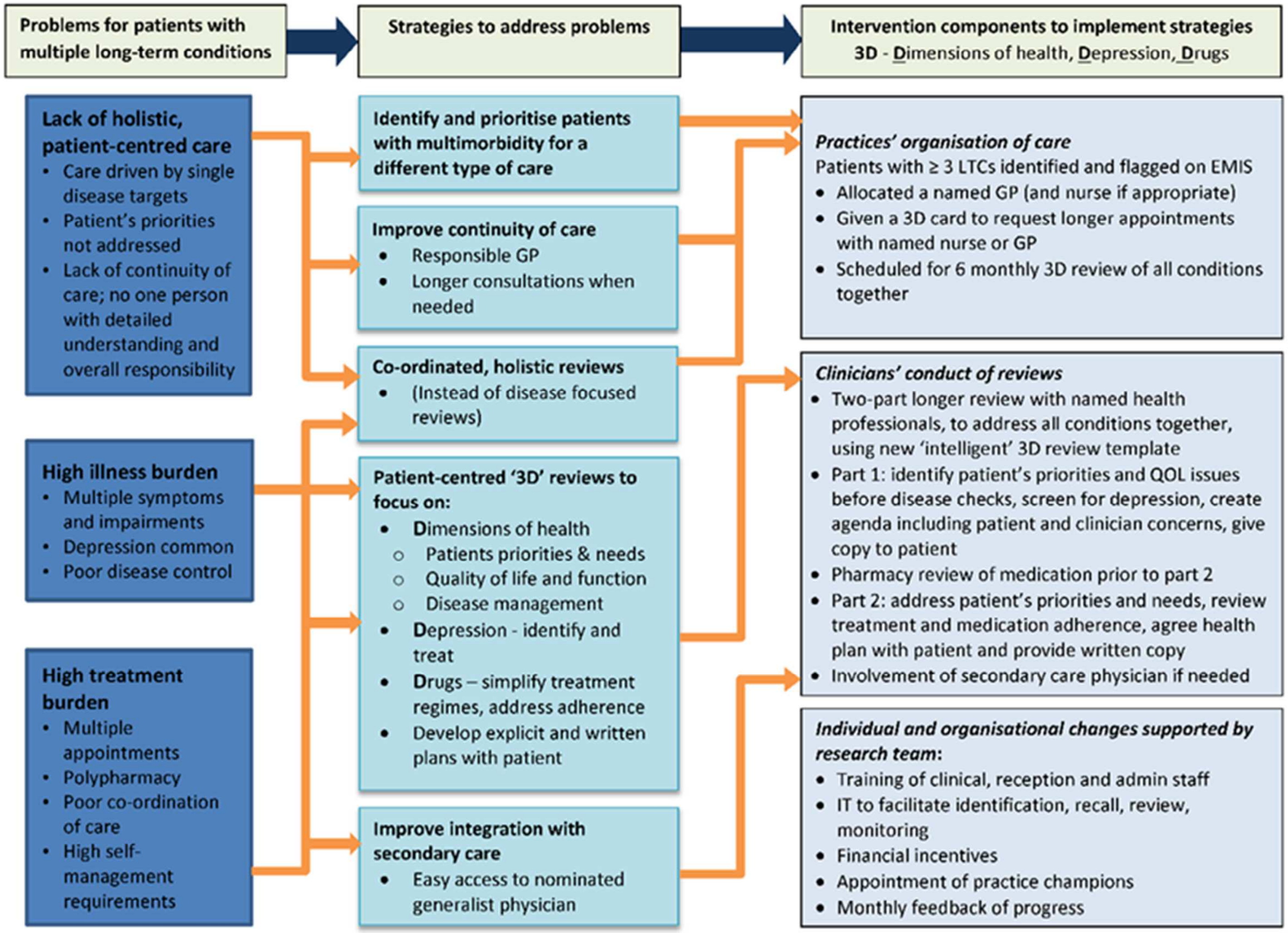

Table 1 Characteristics of participating and non-participating practices

	Participating practices: Bristol (N=12)	Non-participating practices: BNSSG* CCGs (N=86)	Participating practices: Manchester (N=11)	Non-participating practices: Manchester CCGs† (N=181)	All practices: England (N=7674)	Participating practices: Ayrshire & Arran (N=10)	Non-participating practices: Ayrshire & Arran (N=46)	All practices: Scotland (N=982)
**Size**[31,32]								
Average List size	11,360	9,337	8,531	6,389	7,450	6,874	6,869	5,736
**Age profiles**[32,33]								
% aged 65-74	10.3%	8.7%	12.1%	10.9%	17.2%	12.4%	12.1%	10.2%
% aged 75-84	5.8%	5.3%	6.9%	6.1%	7.8%	7.0%	6.9%	5.8%
% aged 85+	2.6%	2.3%	2.9%	2.2%	2.3%	2.6%	2.2%	2.0%
**Deprivation**‡[33,34]								
Deprivation, mean (s.d)	17.3 (13.0)	20.0 (11.3)	14.9 (8.3)	26.5 (11.5)	21.5	28.8 (14.9)	32.5 (15.5)	
**Quality and Outcomes Framework**[21,35]								
QOF achievement (2014/2015)	98.7%	96.6%	96.2%	96.7%	95.5%	99.8%	98.8%	97.3%
**Satisfaction with GP surgery**[36,37]								
Very positive	46.4%	41.9%	50.0%	51.3%	43%	49.1%	47%	87%
Positive	42.4%	44.2%	39.6%	36.8%	42%	39.2%	39%	
Neutral	8.3%	9.4%	7.0%	8.1%	10%	9.8%	12%	10%
Negative	2.9%	4.5%	3.5%	3.8%	5%	1.9%	2%	3%

*BNSSG – Bristol, North Somerset, South Gloucestershire
†Eastern Cheshire, South Cheshire, St Helens, Wigan and Wirral
‡Deprivation is based on IMD 2010 for England and SIMD 2012 for Scotland

Figure 2: Flow of patients into the 3D trial

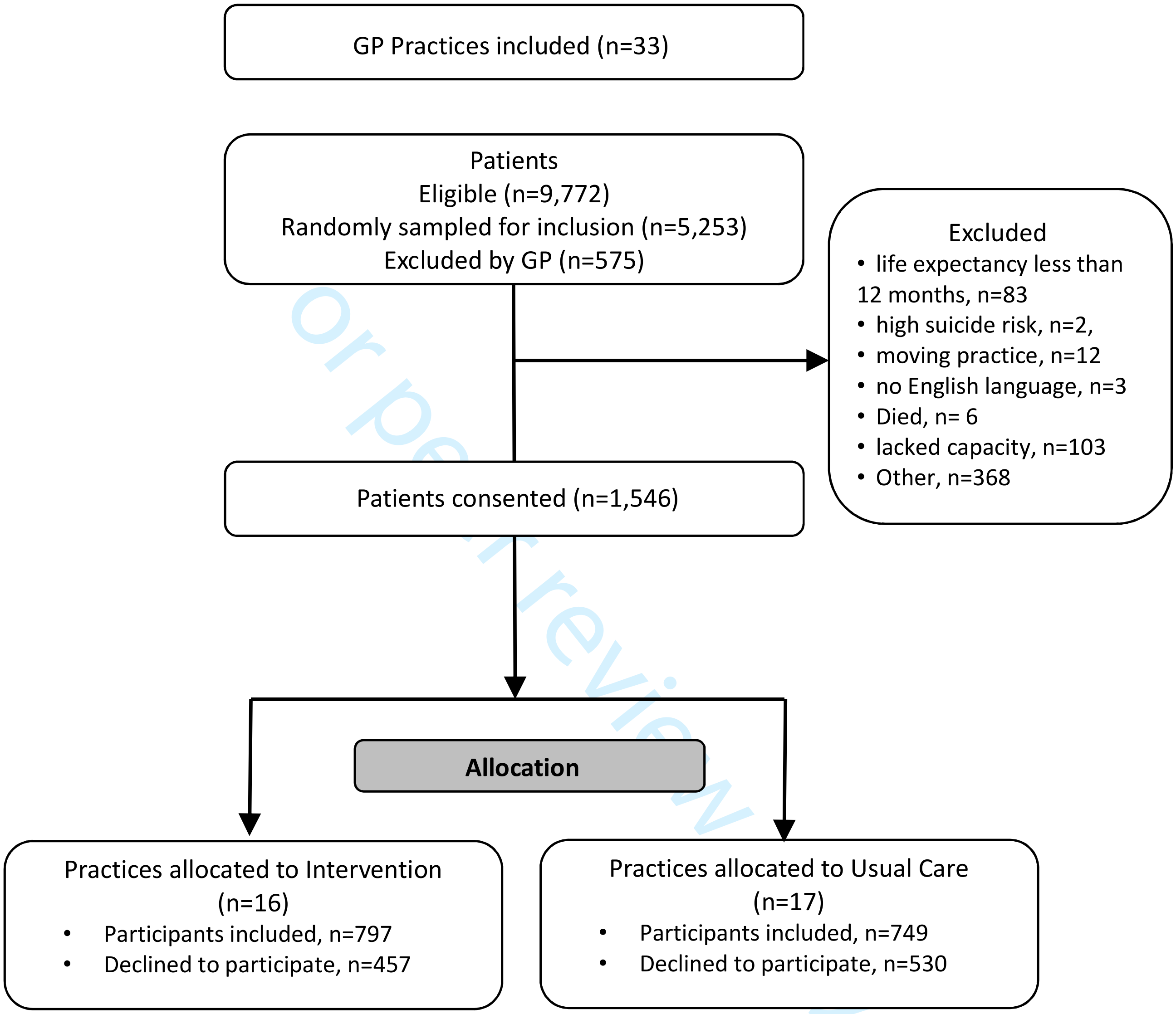

Table 2 Comparison of participating and non-participating patients (long-term conditions on QOF registers, demographic and clinical characteristics)[*]

	Excluded[†] (N=575)	Non-participants[‡] (N=3132)	Participants (N=1546)	Excluded v Invited[§]	Difference between participants and non-participants
Dementia	225 (39%)	340 (11%)	60 (4%)	X^2=456.80, p<0.001	-7% χ^2 =64.39, p<0.001
Depression	246 (43%)	1250 (40%)	559 (36%)	X^2=3.64, p=.057	-4% χ^2 =6.15, p=0.013
Severe Mental Health Group	47 (8%)	200 (6%)	66 (4%)	X^2=5.66, p=.017	-2% χ^2 =8.65, p=0.003
Learning Difficulties	48 (8%)	84 (3%)	14 (1%)	X^2=74.09, p<0.001	-2% χ^2 =15.93, p=0.000
Epilepsy	46 (8%)	186 (6%)	75 (5%)	X^2=5.45, p=.020	-1% χ^2 =1.93, p=0.165
Diabetes	198 (34%)	1616 (52%)	809 (52%)	X^2=62.04, p<0.001	0% χ^2 =0.43, p=0.511
Cardiovascular Disease Group[¶]	521 (91%)	2875 (92%)	1445 (93%)	X^2=2.14, p=.143	+1% χ^2 =4.10, p=0.043
Stroke	215 (37%)	1049 (33%)	528 (34%)	X^2=3.09, p=.079	+1% χ^2 =0.15 p=0.702
Rheumatoid Arthritis	37 (6%)	196 (6%)	103 (7%)	X^2=.0016, p=.968	+1% χ^2 =0.28, p=0.595
Respiratory (asthma or COPD)	191 (33%)	1454 (46%)	772 (50%)	X^2=42.53, p<0.001	+4% χ^2 =4.57, p=0.033
Atrial Fibrillation	164 (29%)	928 (30%)	530 (34%)	X^2=1.68, p=.195	+4% χ^2 =10.44, p=0.001
Male	242 (42%)	1452 (46%)	763 (49%)	X^2=5.70, p=0.017	X^2=3.72, p=0.054
Female	333 (58%)	1680 (54%)	783 (51%)		
Age, mean, (s.d), range	77.14 (14.2) 18-106	71.35 (13.4) 20-101	70.79 (11.5) 25-96	t=10.42, p=<0.001	t=1.40, p=0.163
Morbidity Count, mean (s.d), range	3.39 (0.64) 3-6	3.26 (0.53) 3-7	3.23 (0.48) 3-6	t=6.10, p<0.001	t=2.02, p=0.044
3 co-morbidities	395 (69%)	2444 (78%)	1234 (80%)	X^2=28.98, p<0.001[¥]	X^2=1.96, p=0.161[¥]
4 co-morbidities	140 (24%)	577 (18%)	277 (18%)		
5 co-morbidities	35 (6%)	99 (3%)	31 (2%)		
6 co-morbidities	5 (1%)	11 (0.4%)	4 (0.3%)		
7 co-morbidities		1 (0.03%)			

[*] Since an inclusion criterion was having three or more conditions, the percentages in each column exceed 100%
[†]Eligible on record search but excluded by GP before invitation
[‡]Non-participants combines patients who declined and those who did not respond
[§]Invited includes non-participants & participants combined,
[¶]Includes hypertension, peripheral artery disease, chronic kidney disease, coronary heart disease and/or heart failure
[¥]3 co-morbidities v 4-7 co-morbidities

For peer review only

Table 3 Baseline data on illness and treatment burden (participants)

			Range
Health related Quality of Life - EQ5D (N=1546)	Mean (s.d.)	0.558 (0.287)	-0.51 to1.00
General health (N=1546)	Poor	321 (21%)	
	Fair	681 (45%)	
	Good	429 (28%)	
	Very good	88 (6%)	
	Excellent	5 (0.3%)	
Bayliss (N=1024)	Mean count (s.d.), n	7.5 (3.2), 1543	0 to19
	Mean illness burden* (s.d.), n	18.8 (12.4), 1458	0 to 59
Depression - HADS (N=1512)	Normal	932 (62%)	0 to 7
	Mild	285 (19%)	8 to 10
	Moderate	211 (14%)	11 to 14
	Severe	84 (6%)	15 to 21
Anxiety - HADS (N=1506)	Normal	964 (64%)	0 to 7
	Mild	246 (16%)	8 to 10
	Moderate	204 (14%)	11 to 14
	Severe	92 (6%)	15 to 21
Mean number of medications (self-report)	Mean (s.d.)	8.36 (3.94)	0 to 34
Number of medications (N=1396)	0-4	171 (12%)	
	5-9	781 (56%)	
	10-14	350 (25%)	
	15+	94 (7%)	
Multimorbidity Treatment Burden Questionnaire (N=1524)	None (0)	308 (20%)	
	Low (<10)	385 (25%)	
	Medium (10-22)	425 (28%)	
	High (≥22)	406 (27%)	

*Each self reported health condition is weighted by the extent to which it affects the participants life, from one (not at all) to 5 (a lot)

For peer review only

Table 4 Baseline self-reported data on 'holistic patient centred care'

**Long term condition care**		
	Response	%
Who manages your long term conditions? (N=1436)	GP	920 (64%)
	Nurse	361 (25%)
	Matron	8 (0.6%)
	Hospital doctor	103 (7%)
	Hospital nurse	17 (1%)
How satisfied are you with the care you get at your GP surgery? (N=1494)	Very dissatisfied	36 (2%)
	Fairly dissatisfied	61 (4%)
	Neither	149 (10%)
	Fairly satisfied	489 (33%)
	Very satisfied	759 (51%)
Is the support you receive joined up? (N=1479)*	Not at all	174 (12%)
	Rarely	165 (11%)
	Some of the time	590 (40%)
	Always	550 (37%)
PACIC total (N=1232)†	Mean (s.d.), range 0-5	2.5 (1.0)
Patient activation (N=1454)†	Mean (s.d.), range 1-5	3.0 (1.2)
Decision support (N=1452)†	Mean (s.d.), range 1-5	2.9 (1.0)
Goal setting (N=1443)†	Mean (s.d.), range 1-5	2.3 (1.1)
Problem solving (N=1445)†	Mean (s.d.), range 1-5	2.7 (1.2)
Follow-up / coordination (N=1432)†	Mean (s.d.), range 1-5	2.2 (1.0)
**Continuity of care**		
Do you have a preferred GP? (N=1522)	Yes	1148 (75%)
If yes, how frequently do you see your preferred GP? (N=1141)	Always	508 (44.5%)
	A lot	246 (21.5%)
	Some	294 (26%)
	Never	81 (7%)
	Not tried	8 (0.7%)
	N/A	4 (0.3%)
Asked how my consultations with other doctors going (N=1399)†	Almost never / generally not<br>Sometime<br>Most of time / almost always	888 (63%)<br>229 (16%)<br>282 (20%)
**Whole Person Care**		
GP being interested in you as a whole person (N=1529)‡	Poor	47 (3%)
	Fair	161 (11%)
	Good	284 (19%)
	Very good	449 (29%)
	Excellent	563 (37%)
Nurse being interested in you as a whole person (N=1295)‡	Poor	22 (2%)
	Fair	99 (8%)
	Good	265 (20%)
	Very good	390 (30%)

For peer review only

	Excellent	453 (35%)
**Patient agenda**		
In the last 12 months did you discuss what was most important for you in managing your own health? (N=1479)*	Not at all	259 (18%)
	Rarely	251 (17%)
	Sometimes	520 (35%)
	Always	449 (30%)
Asked how my long term condition affects my life (N=1412)†	Almost never / generally not<br>Sometimes<br>Most of time / almost always	706 (50%)<br>321 (23%)<br>385 (27%)
**Care plans**		
Do you have a written care plan? (N=1526)	No	1112 (73%)
	Yes	151 (10%)
	Don't know	263 (17%)
I was given copy of my plan (N=1410)†	Almost never / generally not<br>Sometimes<br>Most of time / almost always	1055 (75%)<br>131 (9%)<br>224 (16%)
Make a plan that I can do in my daily life (N=1425)†	Almost never / generally not<br>Sometimes<br>Most of time / almost always	829 (58%)<br>223 (16%)<br>373 (26%)

*Taken from LTC6 measure, †Taken from PACIC measure ‡Taken from CARE measure

For peer review only

Table 5 Clinicians' views on care for people with multimorbidity (n=154 from 33 practices)

	Total
Patients with multimorbidity have a special need for holistic, patient-centred care	136 (88%)
Holistic, patient-centred care is enhanced by continuity of care	148 (96%)
Patients being reviewed for a long-term condition should be given a written care plan	96 (62%)
Patients' main concerns may be overlooked during review of long-term conditions	88 (57%)
Patients with 3 or more conditions need longer appointments to address all their concerns	143 (93%)

N (%) of clinicians who agree/strongly agree

For peer review only

Table 6: Results of practice proforma at baseline

Question	Yes<br>N (%)	Comments
Is it your policy to encourage all patients to see their named GP whenever possible?	10 (30)	In most practices, patient request and GP availability determined whether they saw their usual GP. In most of the 10 practices saying 'yes' and in many saying 'no' it was practice policy to fulfil the patient request where possible. However, 1 practice had a formal personal list system ensuring patients saw their own GP.
Is it your policy that every patient with a long-term condition has a face-to-face medication review at least once a year?	25 (76)	This could be with GP, pharmacist or nurse prescriber
Is it your policy that every patient with ≥ 2 long-term conditions receives a written care plan?	1 (3)	Most practices said they used care plans for some conditions (most commonly COPD, diabetes, learning disabilities and dementia). Other conditions included severe mental health conditions, rheumatoid arthritis, various cardiovascular conditions and epilepsy. Only 1 practice said they did not use them for any of the 15 conditions listed and 3 said they only used them for 1 condition. What practices understood by 'care plan' varied and some distinguished between care plans and self-management plans suggesting that they saw care plans as information primarily for health professionals
Is it your formal policy to annually screen for depression all patients with ≥ 2 long-term conditions who are under regular review?	12 (36)	For those answering 'yes' we checked if they routinely used a formal measure such as PHQ2 or PHQ9 for their screening and only counted it as 'yes' if they did.
Is it your policy to offer combined reviews for some patients with multi-morbidity?	31 (94)	11 practices were offering fully combined reviews which meant they were pre-planned, encompassing all LTCs, and both clinician and patient were aware all conditions were to be reviewed. The other 20 either combined a subset of conditions or tried (as far as time and skills allowed) to combine reviews. The remaining 2 were conducting separate reviews.

All answers are reports from the key informant in the practice who was usually a senior administrator or practice manager who could consult with clinical colleagues for answers to some questions. Where possible, when there was ambiguity, answers were clarified by follow-up phone calls.

References

1. National Guideline Centre. Multimorbidity: clinical assessment and management. London: National Institute for Health and Care Excellence, 2016.
2. Barnett K, Mercer S, Norbury M, et al. The epidemiology of multimorbidity in a large cross-sectional dataset: implications for health care, research and medical education. *Lancet* 2012;380:37-43.
3. Salisbury C, Johnson LR, Purdy S, et al. Epidemiology and impact of multimorbidity in primary care: a retrospective cohort study. *Br J Gen Pract* 2011;61(582):e12-e21. doi: 10.3399/bjgp11X548929
4. Howe A. Medical generalism. Why expertise in whole person medicine matters: RCGP, 2012:1-68.
5. Naylor C, Parsonage M, McDaid D, et al. Long-term conditions and mental health: the cost of co-morbidities. London: The King's Fund, 2012.
6. Smith SM, Soubhi H, Fortin M, et al. Managing patients with multimorbidity: systematic review of interventions in primary care and community settings. *BMJ* 2012;345:e5205. doi: 10.1136/bmj.e5205 [published Online First: 2012/09/05]
7. Smith SM, Wallace E, O'Dowd T, et al. Interventions for improving outcomes in patients with multimorbidity in primary care and community settings. *Cochrane Database of Systematic Reviews* 2016(3) doi: 10.1002/14651858.CD006560.pub3
8. Man MS, Chaplin K, Mann C, et al. Improving the management of multimorbidity in general practice: protocol of a cluster randomised controlled trial (The 3D Study). *Bmj Open* 2016;6(4):e011261. doi: 10.1136/bmjopen-2016-011261
9. Stewart M. Towards a global definition of patient centred care. *BMJ* 2001;322(7284):444-45. doi: 10.1136/bmj.322.7284.444
10. American Geriatric Society Expert Panel. Patient-centered care for older adults with multiple chronic conditions: a stepwise approach from the american geriatrics society: american geriatrics society expert panel on the care of older adults with multimorbidity. *J Am Geriatr Soc* 2012;60(10):1957-68. doi: 10.1111/j.1532-5415.2012.04187.x [published Online First: 2012/09/22]
11. Mead N, Bower P. Patient-centredness: a conceptual framework and review of the empirical literature. *Soc Sci Med* 2000;51(7):1087-110. [published Online First: 2000/09/27]
12. Craig P, Dieppe P, Macintyre S, et al. Developing and evaluating complex interventions: the new Medical Research Council guidance. *BMJ* 2008;337:a1655.
13. Loudon K, Treweek S, Sullivan F, et al. The PRECIS-2 tool: designing trials that are fit for purpose. *BMJ : British Medical Journal* 2015;350 doi: 10.1136/bmj.h2147
14. van Deudekom FJ, Postmus I, van der Ham DJ, et al. External validity of randomized controlled trials in older adults, a systematic review. *PloS one* 2017;12(3):e0174053.
15. Worsley SD, Rengerink KO, Irving E, et al. Challenges in Pragmatic Trials: Selection and Inclusion of Usual Care Sites. *Journal of Clinical Epidemiology* 2017
16. Zigmond AS, Snaith RP. The hospital anxiety and depression scale. *Acta Psychiatr Scand* 1983;67(6):361-70. [published Online First: 1983/06/01]
17. EuroQol Group. EuroQol Group EQ-5D-5L [Available from: http://www.euroqol.org/about-eq-5d/valuation-of-eq-5d/eq-5d-5l-value-sets.html accessed 11/02/2015.
18. Bayliss EA, Ellis JL, Steiner JF. Seniors' self-reported multimorbidity captured biopsychosocial factors not incorporated into two other data-based morbidity measures. *J Clin Epidemiol* 2009;62(5):550-57.e1. doi: 10.1016/j.jclinepi.2008.05.002
19. Glasgow RE, Wagner EH, Schaefer J, et al. Development and validation of the Patient Assessment of Chronic Illness Care (PACIC). *Med Care* 2005;43(5):436-44. [published Online First: 2005/04/20]

20. Mercer SW, McConnachie A, Maxwell M, et al. Relevance and practical use of the Consultation and Relational Empathy (CARE) Measure in general practice. *Fam Pract* 2005;22(3):328-34. doi: 10.1093/fampra/cmh730
21. Digital N. Quality and Outcomes Framework 2015 [Available from: http://content.digital.nhs.uk/catalogue/PUB18887 accessed 15/09/2017.
22. Kennedy A, Bower P, Reeves D, et al. Implementation of self management support for long term conditions in routine primary care settings: cluster randomised controlled trial. *Bmj* 2013;346:f2882.
23. Coventry P, Lovell K, Dickens C, et al. Integrated primary care for patients with mental and physical multimorbidity: cluster randomised controlled trial of collaborative care for patients with depression comorbid with diabetes or cardiovascular disease. *bmj* 2015;350:h638.
24. Gallacher KI, Batty GD, McLean G, et al. Stroke, multimorbidity and polypharmacy in a nationally representative sample of 1,424,378 patients in Scotland: implications for treatment burden. *BMC medicine* 2014;12(1):151.
25. May C, Montori VM, Mair FS. We need minimally disruptive medicine. *BMJ* 2009;339 doi: 10.1136/bmj.b2803
26. Rosbach M, Andersen JS. Patient-experienced burden of treatment in patients with multimorbidity–A systematic review of qualitative data. *PloS one* 2017;12(6):e0179916.
27. Burt J, Campbell J, Abel G, et al. Improving patient experience in primary care: a multimethod programme of research on the measurement and improvement of patient experience. 2017
28. Wagner EH, Austin BT, Von KM. Improving outcomes in chronic illness. *Manag Care Q* 1996;4(2):12-25.
29. Rick J, Rowe K, Hann M, et al. Psychometric properties of the patient assessment of chronic illness care measure: acceptability, reliability and validity in United Kingdom patients with long-term conditions. *BMC health services research* 2012;12(1):293.
30. Reeves D, Hann M, Rick J, et al. Care plans and care planning in the management of long-term conditions in the UK: a controlled prospective cohort study. *Br J Gen Pract* 2014;64(626):e568-e75.
31. Digital N. Numbers of Patients Registered at a GP Practice 2015 [Available from: http://content.digital.nhs.uk/catalogue/PUB17356 accessed 05/07/2017.
32. Scotland I. GP workforce and practice list sizes 2015 [Available from: http://www.isdscotland.org/Health-Topics/General-Practice/Publications/data-tables.asp accessed 15/09/2017.
33. England PH. National General Practice Profiles 2015 [Available from: https://fingertips.phe.org.uk/profile/general-practice
34. Scottish Index of Mulitple Deprivation 2012 [Available from: http://geoconvert.mimas.ac.uk/ accessed 15/09/2017.
35. Scotland I. Quality and Outcomes Framework 2015 [Available from: https://www.isdscotland.org/Health-Topics/General-Practice/Quality-And-Outcomes-Framework/2014-15/practicelevel-summaries.asp accessed 15/09/2017.
36. England N. GP patient survey Practice Report 2016 [Available from: https://gp-patient.co.uk/practices-search accessed 14/03/2017.
37. Scotland N. Health and Care Experience Survey 2016 [Available from: http://www.hace15.quality-health.co.uk/index.php/reports/ accessed 15/09/2017.

For peer review only

Appendix 1 Chronic conditions for inclusion

Included patients have three or more diagnoses from the following groups of chronic conditions:

- Cardiovascular disease or Chronic kidney disease (including coronary heart disease, hypertension, heart failure, peripheral arterial disease, chronic kidney disease stage 3 to 5)*
- Stroke
- Diabetes
- Chronic Obstructive Pulmonary Disease or Asthma*
- Epilepsy
- Atrial fibrillation
- Severe mental health problems (schizophrenia or psychotic illness)*
- Depression
- Dementia
- Learning disability
- Rheumatoid arthritis

*Groups are counted only once even if a patient has multiple conditions within a group. For example, having both hypertension and heart failure would just count for one condition

For peer review only

BMJ Open

Understanding usual care for patients with multimorbidity: baseline data from a cluster randomised trial of the 3D intervention in primary care

Journal:	*BMJ Open*
Manuscript ID	bmjopen-2017-019845.R1
Article Type:	Research
Date Submitted by the Author:	29-May-2018

Complete List of Authors:	Chaplin, Katherine ; University of Bristol Centre for Academic Primary Care, Population Health Sciences, Bristol Medical School,<br>Bower, Peter; University of Manchester, NIHR School for Primary Care Research, Centre for Primary Care, Division of Population of Health, Health Services Research and Primary Care, Manchester Academic Health Science Centre<br>Man, Mei-See; University of Bristol Centre for Academic Primary Care, Population Health Sciences, Bristol Medical School, ; University of Bristol, Bristol Randomised Trials Collaboration (BRTC), Population Health Sciences, Bristol Medical School<br>Brookes, Sara ; University of Bristol, Bristol Randomised Trials Collaboration (BRTC), Population Health Sciences, Bristol Medical School<br>Gaunt, Daisy; University of Bristol, Bristol Randomised Trials Collaboration (BRTC), Population Health Sciences, Bristol Medical School<br>Guthrie, Bruce; University of Dundee, Population Health Sciences Division, School of Medicine,<br>Mann, Cindy; University of Bristol Centre for Academic Primary Care, Population Health Sciences, Bristol Medical School,<br>Mercer, Stewart; University of Glasgow, Institute of Health and Wellbeing<br>Rafi, Imran; Royal College of General Practitioners, Clinical Innovation and Research<br>Shaw, Ali; University of Bristol Centre for Academic Primary Care, Population Health Sciences, Bristol Medical School,<br>Salisbury, Chris; University of Bristol Centre for Academic Primary Care, Population Health Sciences, Bristol Medical School,

**Primary Subject Heading**:	General practice / Family practice
Secondary Subject Heading:	Health services research, Patient-centred medicine
Keywords:	Chronic disease, Family practice, Multimorbidity, Patient centred care, Comorbidity

SCHOLARONE™
Manuscripts

For peer review only

TITLE: Understanding usual care for patients with multimorbidity: baseline data from a cluster randomised trial of the 3D intervention in primary care

Katherine Chaplin[1], Peter Bower[2], Mei-See Man[1&3], Sara T Brookes[3], Daisy Gaunt[3], Bruce Guthrie[4], Cindy Mann[1], Stewart Mercer[5], Imran Rafi[6], Alison Shaw[1], Chris Salisbury[1]

Corresponding author:

Prof Chris Salisbury,
Centre for Academic Primary Care, Population Health Sciences, Bristol Medical School
Canynge Hall, 39 Whatley Road,
University of Bristol, Bristol, UK
c.salisbury@bristol.ac.uk
Tel 0117 3314543

Co-authors:

[1] Centre for Academic Primary Care, Population Health Sciences, Bristol Medical School, University of Bristol, Bristol, UK

[2] NIHR School for Primary Care Research, Centre for Primary Care, Division of Population of Health, Health Services Research and Primary Care, Manchester Academic Health Science Centre, University of Manchester, Manchester, UK

[3] Bristol Randomised Trials Collaboration (BRTC), Population Health Sciences, Bristol Medical School, University of Bristol, Bristol, UK

[4] Population Health Sciences Division, School of Medicine, University of Dundee, Dundee, UK

[5] Institute of Health and Wellbeing, General Practice and Primary Care, University of Glasgow, 1 Horslethill Road, Glasgow G12 9LX

[6] Clinical Innovation and Research, Royal College of General Practitioners, Euston Square, London, NW1 2FB

Keywords (up to 5): Chronic disease. Family practice. Multimorbidity. Patient centred care. Comorbidity
Word count (excl title page, abstract & references) 4110
Number of figures = 2
Number of tables/boxes = 6
Number of supplementary files = 4
Number of references = 45

Abstract

Objectives

Recent evidence has highlighted the high prevalence and impact of multimorbidity, but the evidence base for improving management is limited. We have tested a new complex intervention for multimorbidity (the 3D model). The paper describes the baseline characteristics of practices and patients in order to establish the external validity of trial participants. It also explores current 'usual primary care' for multimorbidity, against which the 3D intervention was tested.

Design

Analysis of baseline data from patients in a cluster-randomised controlled trial and additional data from practice staff

Setting

Primary care in the United Kingdom

Participants

Patients with multimorbidity (n=5253), and 154 practice staff

Primary and secondary outcome measures

Using surveys and routinely available data, we compared the characteristics of participating and non-participating practices and participating and non-participating eligible patients.

Baseline questionnaire data from patient participants was used to examine participant illness burden, treatment burden and perceptions of receiving patient-centred care. We obtained data about usual care pre-intervention from practice staff using questionnaires and a structured proforma.

Results

Participating practices were slightly larger, in less deprived areas, and with slightly higher scores for patient satisfaction compared with non-participating practices. Patients with dementia or learning difficulties were likely to be excluded by their GPs, but comparison of participants with non-participants identified only minor differences in characteristics, suggesting that the sample was otherwise representative. Patients reported substantial illness burden, and an important minority reported high treatment burden. Although patients reported relatively high levels of satisfaction with care, many reported not having received potentially important components of care.

Conclusion

This trial achieved good levels of external validity. Although patients were generally satisfied with primary care services, there was significant room for improvement in important aspects of care for multimorbidity that are targeted by the 3D intervention.

Trial registration
Current Controlled Trials ISRCTN06180958

For peer review only

298/300 words

For peer review only

Strengths and limitations of this study

- Data on the external validity of trial populations is often not available, but recruitment using routine GP records allowed us to compare participants and non-participants
- We collected detailed data on care for multimorbidity using validated scales, complemented with data from staff for a more comprehensive assessment.
- Comparisons of participants and non-participants were limited to data available in routine records
- Data on delivery and quality of care were generally based on patient and clinician self-report.

For peer review only

Introduction

Recent evidence has highlighted the importance of multimorbidity for current health policy.[1] Multimorbidity among long-term conditions is the norm among older patients, and is common at a younger age in deprived populations.[2 3] It is associated with significant impacts on quality of life, mortality, and health care utilisation.[1]

There is increasing consensus on the sort of care that is required for the management of patients with multimorbidity.[1 4 5] Much of this derives from consensus about high quality care for long-term conditions more generally, with a focus on care planning, shared decision-making, and self-management.[1 6-8] However, management of patients with multimorbidity also raises specific challenges, such as how to prioritise among conditions, and how best to manage the treatment burden experienced due to multiple treatments and multiple appointments.[7 9] The increased prevalence of depression in multimorbidity is well recognised, and comorbid depression is associated with worse outcomes.[10]

However, the evidence base for the management of multimorbidity remains sparse. A recent Cochrane review reported only 18 randomised trials specifically targeting multimorbidity, and concluded that 'there are remaining uncertainties about the effectiveness of interventions for people with multimorbidity in general due to the relatively small number of RCTs conducted in this area to date.[6 11] The National Institute for Health and Care Excellence (NICE - the leading UK organisation for the development of clinical guidelines) has published guidelines for the clinical assessment and management of multimorbidity, reviewing the evidence for varying 'format of encounters' in people with multimorbidity (including longer consultations, structured recall, involving the patient in agenda-setting, and multi-professional appointments) and for primary care based comprehensive geriatric assessment.[1] However, the evidence available did not support any specific recommendation on how to organise primary care to better meet the needs of people with multimorbidity. Instead the guideline development group recommended that trials were needed evaluating new organisational approaches for people with multimorbidity.

The Cochrane review suggested that, given the complexity of needs and management of patients with multimorbidity, interventions are likely to be 'complex' (i.e. 'involving several components acting in concert to improve care').[6 11] Our team has developed the 3D model for the management of multimorbidity in primary care. The model is described in full elsewhere,[12] and has recently undergone evaluation in a large scale randomised controlled trial (ISRCTN06180958) with concurrent economic and process evaluation.[12 13]

Key problems posed by current healthcare organisation and experienced by patients with multimorbidity are a lack of holistic patient-centred care, a high burden of illness and a high level of treatment burden due to multiple medications and the need to attend numerous appointments. Figure 1 shows how the 3D approach addresses these problems. The basis for 3D is the patient-centred care model,[5 14 15] which includes four components:

- A focus on the patient's individual disease and illness experience
- A biopsychosocial perspective
- Finding common ground on what the problem is and mutually agreeing management plans
- Enhancing the relationship between the patient and doctor (the therapeutic alliance)

<Insert Figure 1: 3D logic model including theoretical mechanisms of action>

The Medical Research Council has a well-developed framework for the development, evaluation and description of complex interventions.[16] Recent work in this area has also emphasised two additional issues. First, there is a need to understand the practice and patient populations who actually enter trials of complex interventions, compared to those who are potentially eligible, to better understand the external validity of the study.[17 18] Secondly, there is a need to better understand the comparator to the intervention (in this case, 'usual primary care') in order to understand the content and quality of care against which the complex intervention is being tested.[19] The aims of this study are therefore to:

1. Compare practices and patients participating in the trial with non-participants

2. Describe the characteristics of participating patients at baseline in terms of their experiences of (a) illness burden (b) treatment burden and (c) patient centred care

3. Describe usual care for people with multimorbidity

For peer review only

Methods

Design

The design of the 3D trial and process evaluation has been described in full[12 13] and is briefly summarised here. The 3D trial is a multi-centre pragmatic, two-arm, practice-level cluster randomised controlled trial. In the United Kingdom, each patient is registered to receive free health care under the National Health Service (NHS) from one local general practice. For most patients, chronic disease management is provided by general practitioners (GPs) and nurses within their registered practice with little or no involvement from hospital specialists. Practices are incentivised to provide high quality care for many specified chronic diseases by the NHS Quality and Outcomes Framework (QOF) pay-for-performance scheme.[20]

The 3D study is based in general practices in three areas; Bristol and Greater Manchester in England and Ayrshire in Scotland. Volunteer practices were recruited from areas with a range of socioeconomic characteristics. For inclusion practices had to have at least 2 GPs and 4500 registered patients and to use the EMIS clinical IT system (used by the majority of practices in the UK). Inclusion criteria for patients were age 18+ and having three or more types of long-term condition from those included in the QOF (Appendix A). We decided to include patients with three or more (rather than two or more) conditions in order to focus effort on more complex patients who may have more to gain from a new model of organisation. Up to 150 potentially eligible patients were randomly selected from each practice by a researcher using a random number function in Microsoft Excel, and using an anonymous patient identifier. Selected patients' notes were screened by their GPs against the following exclusion criteria: having a life expectancy of less than 12 months; serious suicidal risk; known to be leaving the practice within 12 months; unable to complete questionnaires in English even with the help of carers; actively taking part in other research involving extra visits to primary care or other health services; lacking capacity to consent (Scotland only); or being considered unsuitable for the research study by their GP. All remaining patients were sent an invitation from their practice including information about the study, a consent form and baseline questionnaire. Patients self-consented by returning the consent form and completed baseline questionnaire to the research team, using a freepost envelope. Non-respondents were sent one postal reminder 10-14 days later, supplemented by a telephone reminder in those practices where recruitment targets were not met.

Patient data

We had data on two groups of patients. For patients who were invited to the trial ('potentially eligible patients'), we had data on age, sex and QOF-recorded conditions.

For eligible patients who consented to take part ('participating patients'), data was also available from the baseline questionnaire measuring depression (Hospital Anxiety and Depression Scale)[21], quality of life (EQ5D-5L)[22], illness burden (Bayliss)[23], treatment burden (MTBQ)[24], patients' perception of the quality of chronic illness care (PACIC)[25] and perceived empathy of GPs and nurses (CARE).[26]

The patient questionnaire included several questions about holistic patient centred care. These included the PACIC measure and the CARE measure, along with two questions from the Long Term Conditions 6 (LTC6) questionnaire.[27] Three further questions were included regarding satisfaction with current care, whether patients usually saw their preferred GP, and whether they had a written care plan (all based on the national GP Patient Survey).[28]

For peer review only

Staff perceptions and practice data on the organisation of care

At the start of the trial, participating GPs and practice nurses completed a purpose-designed questionnaire about their beliefs and attitudes regarding care of patients with multimorbidity. Researchers training the nurses and GPs in intervention practices asked them to complete the questionnaire before the training began. In usual care practices the questionnaire was distributed via the practice manager and followed up with one researcher reminder where there was a poor response. The questionnaire consisted of 12 statements that were scored from 1 ('strongly disagree') to 5 ('strongly agree') (Appendix B). Only those questions which can be compared with patient's perspectives have been reported.

In addition, information about how the practice organised usual care for patients with long-term conditions was collected from all practices through a structured proforma completed by a single key respondent in each practice (usually the practice manager) via an emailed survey supplemented by telephone or face-to-face interview. This covered staff resources, organisation of long term condition review clinics and practice policy on medication reviews, care plans and continuity of care.

Analysis

In order to compare practices and patients participating in the trial with non-participants, we compared the characteristics of practices in the 3D trial with practices in the same Clinical Commissioning Group (CCG) and national data. We assessed differences in patient populations (age, deprivation), practice size, and published assessments of quality (the percentage of targets met within the QOF)[29] and patient satisfaction (based on the national GP Patient Survey).[28]

We described the demographic and clinical characteristics of patients at each stage of recruitment to 3D – those identified as potentially eligible but excluded by their GP, those eligible but not participating (due to non-response or actively declining), and those who agreed to participate in the study. All comparisons were analysed in multi-level regression models which included practice as a random effect. For participants in the trial we present descriptive data on patients self-reported baseline measures of their illness burden and treatment burden. Because the number of potential participants is large (n=5253), we have interpreted whether absolute differences are meaningful rather than relying only on p values (since even small and non-meaningful differences will generate small p values with large samples).

To describe the extent to which current care for people with multimorbidity is patient-centred from the perspective of patients we present participant responses to individual question items from the baseline patient questionnaire reflecting key concepts in patient centred care.

We also provide data about staff views about care for people with multimorbidity and report descriptive data from the structured proforma about usual care for patients with multimorbidity in all practices participating in the trial.

For all descriptive analyses we have calculated intra-cluster correlation co-efficients (ICCs) to demonstrate the extent of practice-level variability. All analyses were performed using Stata V.15 (StataCorp).

For peer review only

Patient and public involvement

An active group of up to 14 patients and carers provided a service user perspective. Through regular meetings with the research team they contributed to refinement of the research questions and design of the intervention. The group were consulted about the perceived burden of the intervention, and provided valuable feedback on the specific outcome measures chosen including helping to develop the measure of treatment burden. They particularly contributed to communications with participants in recruitment materials and regular newsletters about progress. The findings will be available to participants and the public on the trial website.

For peer review only

Results

What types of practices and patients participated in the 3D trial, and how did they compare to non-participants?

Across the 3 sites, 68 practices expressed initial interest in the study, of which 35 signed up to the study. Two practices subsequently withdrew prior to randomisation. The remaining 33 practices (49% of those expressing interest) were randomised, 16 into the intervention arm and 17 to usual care. Descriptive characteristics of the 33 practices are shown in Table 1. Compared with all practices in their local area, practices which agreed to participate tended to be slightly larger, in less deprived areas, and had slightly higher scores for patient satisfaction (Table 1).

For peer review only

Table 1 Characteristics of participating and non-participating practices

	Participating practices: Bristol (N=12)	Non-participating practices: BNSSG* CCGs (N=86)	Participating practices: Manchester (N=11)	Non-participating practices: Manchester CCGs† (N=181)	All practices: England (N=7674)	Participating practices: Ayrshire & Arran (N=10)	Non-participating practices: Ayrshire & Arran (N=46)	All practices: Scotland (N=982)
**Size**[30 31]								
Average List size (s.d.)	11,360 (3,950)	9,337 (3,792)	8,531 (3,768)	6,389 (3,861)	7,450 (NR)§	6,874 (2,813)	6,869 (3,565)	5,736 (3,591)
**Age profiles**[31 32]								
% aged 65-74	10.3%	8.7%	12.1%	10.9%	17.2%	12.4%	12.1%	10.2%
% aged 75-84	5.8%	5.3%	6.9%	6.1%	7.8%	7.0%	6.9%	5.8%
% aged 85+	2.6%	2.3%	2.9%	2.2%	2.3%	2.6%	2.2%	2.0%
**Deprivation**‡[32 33]								
Deprivation, mean (s.d)	17.3 (13.0)	20.0 (11.3)	14.9 (8.3)	26.5 (11.5)	21.5	28.8 (14.9)	32.5 (15.5)	
**Quality and Outcomes Framework**[29 34]								
QOF achievement (2014/2015)	98.7%	96.6%	96.2%	96.7%	95.5%	99.8%	98.8%	97.3%
**Satisfaction with GP surgery**[35 36]								
Very positive	46.4%	41.9%	50.0%	51.3%	43%	49.1%	47%	87%
Positive	42.4%	44.2%	39.6%	36.8%	42%	39.2%	39%	
Neutral	8.3%	9.4%	7.0%	8.1%	10%	9.8%	12%	10%
Negative	2.9%	4.5%	3.5%	3.8%	5%	1.9%	2%	3%

*BNSSG – Bristol, North Somerset, South Gloucestershire

†Eastern Cheshire, South Cheshire, St Helens, Wigan and Wirral

‡Deprivation is based on IMD 2010 for England and SIMD 2012 for Scotland

For peer review only - http://bmjopen.bmj.com/site/about/guidelines.xhtml

The flow of patients into the trial is shown in Figure 2. Between 20 May 2015 and 31 December 2015, a total of 9772 patients were identified as potentially eligible, representing 3.9% of the adult population. Of these, 5253 were randomly sampled from practice registers. GPs excluded 575 (11%) of those based on medical record data because they were ineligible or the GP felt it would be inappropriate to invite them to participate. Potential participants who were excluded by their GPs were more likely to have dementia or learning difficulties and less likely to have diabetes or respiratory conditions than those not excluded (Table 2). There was considerable variation between practices in the percentage of patients excluded (mean=11.01%, s.d.=8.01%).

<Insert Figure 2: Flow of patients into the 3D trial>

Of 4678 patients invited to participate, 1546 (33%) provided consent. Differences between participants and non-participants in terms of their health conditions were small, except that participants were less likely to have dementia than non-participants. Of the 11 types of long-term condition which made people eligible for the trial the most commonly reported were cardiovascular disease (including hypertension, peripheral artery disease, chronic kidney disease, coronary heart disease and heart failure; affecting 93% of participants), diabetes (52%) and respiratory conditions (asthma or chronic obstructive pulmonary disease; 50%).

Baseline demographic and health data on excluded patients, non-participants and participants are shown in Table 2. Excluded patients were more likely to be female, older and have 4 or more conditions than those invited. Participants and non-participants had very similar demographic characteristics and experienced a similar number of health conditions.

Table 2 Comparison of participating and non-participating patients (long-term conditions on QOF registers, demographic and clinical characteristics)*

	Excluded† (N=575)	Non-participants‡ (N=3132)	Participants (N=1546)	Excluded v Invited§¶	Difference between participants and non-participants¶
Dementia	225 (39%)	340 (11%)	60 (4%)	OR=0.12, p<0.001	-7% OR=0.32, p<0.001
Depression	246 (43%)	1250 (40%)	559 (36%)	OR=0.83, p=0.037	-4% OR=0.87, p=0.037
Severe Mental Health Group	47 (8%)	200 (6%)	66 (4%)	OR=0.66, p=0.014	-2% OR=0.66, p=0.004
Learning Difficulties	48 (8%)	84 (3%)	14 (1%)	OR=0.22, p<0.001	-2% OR=0.33, p<0.001
Epilepsy	46 (8%)	185 (6%)	76 (5%)	OR=0.68, p=0.021	-1% OR=0.81, p=0.128
Diabetes	198 (34%)	1613 (52%)	812 (53%)	OR=2.07, p<0.001	0% OR=1.03, p=0.641
Cardiovascular Disease Group¥	521 (91%)	2875 (92%)	1445 (93%)	OR=1.30, p=0.091	+1% OR=1.25, p=0.066
Stroke or TIA	215 (37%)	1050 (34%)	527 (34%)	OR=0.87, p=0.124	+1% OR =1.02 p=0.741
Rheumatoid Arthritis	37 (6%)	196 (6%)	103 (7%)	OR=0.99, p=0.964	+1% OR=1.06, p=0.631
Respiratory	191 (33%)	1456 (46%)	770 (50%)	OR=1.87,	+4%

For peer review only

(asthma or COPD)				p<0.001	OR=1.21, p=0.003
Atrial Fibrillation	164 (29%)	928 (30%)	530 (34%)	OR=1.17, p=0.114	+4% OR=1.19, p=0.009
Male	242 (42%)	1452 (46%)	763 (49%)	OR=0.81, p=0.018	OR=0.90, p=0.078
Age, mean, (s.d), range	77.14 (14.2) 18-106	71.35 (13.4) 20-101	70.79 (11.5) 25-96	β= -6.04, p<0.001	β= -1.11, p=0.005
Morbidity Count, mean (s.d), range	3.39 (0.64) 3-6	3.26 (0.53) 3-7	3.23 (0.48) 3-6	β= -0.14, P<0.001	β= -0.03, p=0.044
3 co-morbidities	395 (69%)	2444 (78%)	1234 (80%)	OR=1.66, p<0.001[#]	OR=1.12, p=0.148[#]
4 co-morbidities	140 (24%)	577 (18%)	277 (18%)		
5 co-morbidities	35 (6%)	99 (3%)	31 (2%)		
6 co-morbidities	5 (1%)	11 (0.4%)	4 (0.3%)		
7 co-morbidities		1 (0.03%)			

[*] Since an inclusion criterion was having three or more conditions, the percentages in each column exceed 100%
[†] Eligible on record search but excluded by GP before invitation
[‡] Non-participants combines patients who declined and those who did not respond
[§] Invited includes non-participants & participants combined
[¶] ORs were calculated using a multi-level logistic regression with practice included as a random effect, β coefficients were calculated using a multi-level linear regression with practice included as a random effect
[¥] Includes hypertension, peripheral artery disease, chronic kidney disease, coronary heart disease and/or heart failure
[#] 3 co-morbidities v 4-7 co-morbidities

Baseline characteristics of participating patients in terms of (a) illness burden and (b) treatment burden

Two thirds of patients (66%) reported having fair or poor health, with less than 7% reporting very good or excellent health (Table 3). Although inclusion to the trial was based on QOF conditions in medical records, patients self-reported an average of seven conditions from the more comprehensive list included in the Bayliss measure.[23] Based on the HADs measure, more than a third of patients (38%) reported anxiety or depression of at least mild severity.

Table 3 Baseline data on illness and treatment burden (participants)

			ICC (95% CI)
Health related Quality of Life - EQ5D (N=1546)	Mean (s.d.) , range -0.51-1.00	0.558 (0.287)	0.033 (0.007-0.059)
General health (N=1546)	Poor	321 (21%)	0.034 (0.008-0.060)
	Fair	681 (45%)	
	Good	429 (28%)	
	Very good	88 (6%)	
	Excellent	5 (0.3%)	
Bayliss	Mean count (s.d.), n, range 1-73	7.5 (3.2), 1543	0.003 (0.000-0.014)
	Mean illness burden[*] (s.d.), n, range 1-26	18.8 (12.4), 1458	0.023 (0.001-0.046)
Depression - HADS (N=1512)	Normal (0 to 7)	932 (62%)	0.041 (0.011-0.070)
	Mild (8 to 10)	285 (19%)	
	Moderate (11 to 14)	211 (14%)	

For peer review only

	Severe (15 to 21)	84 (6%)	
Anxiety - HADS (N=1506)	Normal (0 to 7)	964 (64%)	0.029 (0.005-0.053)
	Mild (8 to 10)	246 (16%)	
	Moderate (11 to 14)	204 (14%)	
	Severe (15 to 21)	92 (6%)	
Mean number of medications (self-report)	Mean (s.d.), range 0-34	8.36 (3.94)	0.018 (0.000-0.039)
Number of medications (N=1396)	0-4	171 (12%)	0.018 (0.000-0.039)
	5-9	781 (56%)	
	10-14	350 (25%)	
	15+	94 (7%)	
Multimorbidity Treatment Burden Questionnaire (N=1524)	None (0)	308 (20%)	0.026 (0.003-0.049)
	Low (<10)	385 (25%)	
	Medium (10-22)	425 (28%)	
	High (≥22)	406 (27%)	

*Each self reported health condition is weighted by the extent to which it affects the participants life, from one (not at all) to 5 (a lot)

On average patients reported regularly taking eight medications with 32% of patients taking at least 10 regular medications (Table 4). More than half (55%) reported at least a moderate level of treatment burden, with a score of at least ten on the MTBQ. This score would be achieved, for instance, by having some difficulty in at least two areas of health care, or severe difficulty in at least one area.

Table 4 Baseline self-reported data on 'holistic patient centred care'

	Response	%	ICC (95% CI)
**Long term condition care**			
Who manages your long term conditions? (N=1436)	GP	920 (64%)	0.036 (0.008-0.064)
	Nurse	361 (25%)	0.056 (0.019-0.093)
	Matron	8 (0.6%)	0.001 (0.000-0.013)
	Hospital doctor	103 (7%)	0.006 (0.000-0.021)
	Hospital nurse	17 (1%)	0.000 (0.000-0.012)
How satisfied are you with the care you get at your GP surgery? (N=1494)	Very dissatisfied	36 (2%)	0.067 (0.026-0.108)
	Fairly dissatisfied	61 (4%)	
	Neither	149 (10%)	
	Fairly satisfied	489 (33%)	
	Very satisfied	759 (51%)	
Do you think the support and care you receive is joined up and working for you? (N=1479)*	Not at all	174 (12%)	0.041 (0.011-0.071)
	Rarely	165 (11%)	
	Some of the time	590 (40%)	
	Always	550 (37%)	

For peer review only

PACIC total (N=1232)†	Mean (s.d.), range 0-5	2.5 (1.0)	0.044 (0.011-0.078)
Patient activation (N=1454) †	Mean (s.d.), range 1-5	3.0 (1.2)	0.041 (0.011-0.070)
Decision support (N=1452) †	Mean (s.d.), range 1-5	2.9 (1.0)	0.029 (0.005-0.054)
Goal setting (N=1443) †	Mean (s.d.), range 1-5	2.3 (1.1)	0.029 (0.004-0.053)
Problem solving (N=1445) †	Mean (s.d.), range 1-5	2.7 (1.2)	0.041 (0.011-0.072)
Follow-up / coordination (N=1432) †	Mean (s.d.), range 1-5	2.2 (1.0)	0.035 (0.007-0.062)
**Continuity of care**			
Do you have a preferred GP? (N=1522)	Yes	1148 (75%)	0.038 (0.010-0.065)
If yes, how frequently do you see your preferred GP? (N=1141)	Always	508 (44.5%)	0.127 (0.062-0.192)
	A lot	246 (21.5%)	
	Some	294 (26%)	
	Never	81 (7%)	
	Not tried	8 (0.7%)	
Asked how my consultations with other doctors going (N=1399)†	Almost never / generally not<br>Sometime<br>Most of time / almost always	888 (63%)<br>229 (16%)<br>282 (20%)	0.037 (0.008-0.064)
**Whole Person Care**			
GP being interested in you as a whole person (N=1529)‡	Poor	47 (3%)	0.071 (0.029-0.113)
	Fair	161 (11%)	
	Good	284 (19%)	
	Very good	449 (29%)	
	Excellent	563 (37%)	
Nurse being interested in you as a whole person (N=1295)‡	Poor	22 (2%)	0.027 (0.002-.052)
	Fair	99 (8%)	
	Good	265 (20%)	
	Very good	390 (30%)	
	Excellent	453 (35%)	
**Patient agenda**			
In the last 12 months did you discuss what was most important for you in managing your own health? (N=1479)*	Not at all	259 (18%)	0.017 (0.000-0.036)
	Rarely	251 (17%)	
	Sometimes	520 (35%)	
	Always	449 (30%)	
Asked how my long term condition affects my life (N=1412)†	Almost never / generally not<br>Sometimes<br>Most of time / almost always	706 (50%)<br>321 (23%)<br>385 (27%)	0.036 (0.008-0.064)
**Care plans**			
Do you have a written care plan? (N=1526)	No / Don't know	1375 (90%)	0.008 (0.000-0.023)
	Yes	151 (10%)	

For peer review only

I was given copy of my plan (N=1410)†	Almost never / generally not<br>Sometimes<br>Most of time / almost always	1055 (75%)<br>131 (9%)<br>224 (16%)	0.023<br>(0.000-0.045)
Make a plan that I can do in my daily life (N=1425)†	Almost never / generally not<br>Sometimes<br>Most of time / almost always	829 (58%)<br>223 (16%)<br>373 (26%)	0.027<br>(0.003-0.052)

*Taken from LTC6 measure, †Taken from PACIC measure ‡Taken from CARE measure

The extent to which current care for people with multimorbidity is patient-centred from the perspective of patients

Table 4 shows that most patients indicated that a GP or primary care nurse was responsible for their long-term condition, and reported relatively high levels of overall satisfaction with their care, although reported levels of care co-ordination were somewhat lower. Three quarters had a preferred GP and of these 66% saw that GP 'most of the time'. In terms of 'whole person care', approximately two thirds of patients reported that their GP and nurse were 'excellent' or 'very good' at 'being interested in them as a whole person'. However, only 37% reported that their care was always 'joined up'.

The data show that many patients do not perceive care as patient-centred in terms of focusing on an individual's experience and agreeing management plans. A relatively high proportion of patients (35%) reported 'rarely' or 'not at all' discussing what was most important to them in terms of their health (Table 4). Only 10% of participants reported having a care plan. Scores on the PACIC scale were around the mid-point of the scale, with the highest ratings for 'activation' and 'decision support', and the lowest for 'goal setting' and 'follow up' (Table 4).

The extent to which current care for people with multimorbidity is patient-centred from the perspective of primary care clinicians

The vast majority (88%) of clinicians agreed that patients with multimorbidity have a special need for patient centred care and over 95% agreed that continuity of care improves patient-centred care (Table 5). Most clinicians agreed that patients with a long-term condition should be given a care plan and that they were more likely to adhere to goals they had suggested themselves, but were evenly divided on whether patients preferred the clinician to make the plan. More than half of the clinicians agreed that patients' main concerns may be overlooked in long-term condition reviews (Table 5). Almost all clinicians (93%) felt that patients with multimorbidity need longer appointments to address all their concerns.

Table 5 Clinicians' views on care for people with multimorbidity (n=154 from 33 practices)

	Total	ICC (95% CI)
Patients with multimorbidity have a special need for holistic, patient-centred care	136 (88%)	0.000<br>(0.000-0.116)
Holistic, patient-centred care is enhanced by continuity of care	148 (96%)	0.000<br>(0.000-0.116)
Patients being reviewed for a long-term condition should be given a written care plan	96 (62%)	0.188<br>(0.023-0.352)
Patients' main concerns may be overlooked during review of long-term conditions	88 (57%)	0.037<br>(0.000-0.165)
Patients with 3 or more conditions need longer appointments to address all their concerns	143 (93%)	0.040<br>(0.000-0.167)

N (%) of clinicians who agree/strongly agree

For peer review only

The extent to which current usual care aligns with the 3D model, on the basis of practice policies

Only one of the 33 practices said they routinely provided patients with a written care plan (and 80% of the patients in that practice said they did not have a written care plan). Only one third (n=10) of practices had an active policy to encourage continuity of care, with the majority of others saying they try to accommodate patient preference. Only 36% of practices said they routinely performed depression screening while 76% said they conducted face to face medication reviews at least annually. All except two practices said they tried to combine reviews of some long-term conditions which might lessen treatment burden and improve joined up care (Table 6).

Table 6: Results of practice proforma at baseline

Question	Yes N (%)	Comments
Is it your policy to encourage all patients to see their named GP whenever possible?	10 (30)	In most practices, patient request and GP availability determined whether they saw their usual GP. In most of the 10 practices saying ‘yes’ and in many saying ‘no’ it was practice policy to fulfil the patient request where possible. However, 1 practice had a formal personal list system ensuring patients saw their own GP.
Is it your policy that every patient with a long-term condition has a face-to-face medication review at least once a year?	25 (76)	This could be with GP, pharmacist or nurse prescriber
Is it your policy that every patient with ≥ 2 long-term conditions receives a written care plan?	1 (3)	Most practices said they used care plans for some conditions (most commonly COPD, diabetes, learning disabilities and dementia). Other conditions included severe mental health conditions, rheumatoid arthritis, various cardiovascular conditions and epilepsy. Only 1 practice said they did not use them for any of the 15 conditions listed and 3 said they only used them for 1 condition. What practices understood by ‘care plan’ varied and some distinguished between care plans and self-management plans suggesting that they saw care plans as information primarily for health professionals
Is it your formal policy to annually screen for depression all patients with ≥ 2 long-term conditions who are under regular review?	12 (36)	For those answering ‘yes’ we checked if they routinely used a formal measure such as PHQ2 or PHQ9 for their screening and only counted it as ‘yes’ if they did.
Is it your policy to offer combined reviews for some patients with multi-morbidity?	31 (94)	11 practices were offering fully combined reviews which meant they were pre-planned, encompassing all LTCs, and both clinician and patient were aware all conditions were to be reviewed. The other 20 either combined a subset of conditions or tried (as far as time and skills allowed) to combine reviews. The remaining 2 were conducting separate reviews.

All answers are reports from the key informant in the practice who was usually a senior administrator or practice manager who could consult with clinical colleagues for answers to some questions. Where possible, when there was ambiguity, answers were clarified by follow-up phone calls.

The ICCs reported in tables 3 to 6 suggest low levels of clustering (ICC <0.05) for all outcomes except for some variables relating to practice organisation of care, such as whether care is provided mainly

by nurses or doctors, participant satisfaction with care, and clinicians' attitudes to written care plans.

For peer review only

Discussion

Summary of the findings

The paper describes usual care for people with high-levels of multimorbidity using baseline data from a cohort of patients entering a trial. Comparison of patients entering the trial with non-participants identified only minor differences in demographic and clinical characteristics, suggesting good external validity. As anticipated, participants in the trial reported high levels of illness burden and treatment burden. Although participants reported relatively high levels of satisfaction with their relationships with professionals, responses to specific questions identified important gaps in their experience of care as patient-centred. Although clinicians supported aspects of patient-centred care such as continuity of care and care plans, and claimed to provide these, the experiences of patients were variable. The results of this study suggest that there is significant room for improvement in many aspects of care for multimorbidity that are targeted by the 3D intervention. In particular, the results suggest a need for improvements in the continuity and co-ordination of care, more focus on the problems which matter most to patients (including mental as well as physical health), more effort to reduce the burden of treatment and more attention to goal-setting and sharing written care plans.

Strengths and limitations

A key strength of this study was our ability to collect comparative data on 'potentially eligible' patients, to allow us to compare participants and non-participants. Data on the external validity of trial populations is often not available, but recruitment using routine GP records does provide significant advantages in this regard. We also collected detailed data on care for multimorbidity using validated scales, and complemented these with data from staff to provide a more comprehensive assessment.

Detailed comparisons of participants and non-participants are inevitably difficult because more detailed survey data are by definition not available for non-participants, and comparisons are restricted to basic demographic characteristics. However in this study we have used anonymised practice records to compare clinical diagnoses and been able to show that participants have similar characteristics to non-participants. The bulk of the findings in this study about patient centred aspects of care come from self-report from patients and professionals, and we do not know how these relate to actual delivery of care in these practices. However, a key aim of the intervention is to improve patient experience of care, for which self-report is the optimal assessment method.

As a pragmatic trial, 3D is designed to recruit a population with high external validity by ensuring that practices and patients who participate are representative of the wider population to whom the intervention, if effective, would be provided in real life. The overall response rate among patients invited was 33%. This is likely to be an under-estimate of the proportion of eligible patients recruited because some non-responders may not have been eligible. Nevertheless, this recruitment rate is typical of previous studies in UK populations of primary care patients with long-term conditions,[37 38] and may be considered relatively high given that the inclusion criteria for this trial selected elderly patients with multiple illnesses.

Our inclusion criteria were based on patients with 3 or more types of condition from a list of 17 conditions included in the QOF framework, a pay-for-performance scheme. The use of a wider list of conditions may have led to selection of a different group of patients, but we based our selection on QOF conditions because they are prevalent, clinically important, and reliably coded.

For peer review only

Interpretation of the findings and comparisons with the wider literature

We raised three main issues in this paper. First, how do practices and patients in 3D compare to the wider primary care population outside the trial? Although limited by available data, the comparisons suggested that the consenting sample did not differ markedly from the potentially eligible population on measured characteristics, with the largest difference being the proportion with dementia or learning difficulties, which is unsurprising given the nature of the recruitment method. We sought to ensure that our inclusion criteria were as wide as possible, but this study further demonstrates the difficulty of recruiting patients with dementia and learning difficulties within trials. Our findings suggest that to increase inclusion rates of people with these conditions it is important not only to have strategies to encourage patient participation, but also to address the reluctance of some clinicians to even allow them to be invited to participate. Although we cannot be sure that patients agreeing to take part do not differ on other important characteristics, the data do provide some confidence that the results are not based on a highly selected sample, especially in terms of physical health conditions.

The second issue is the levels of illness burden, treatment burden and patient-centred care experienced by patients with multimorbidity. Our recruitment method used a simple method of condition counts which is easy to conduct, but it was unclear whether we would identify patients with high needs. In terms of illness burden, our data suggest a sample with relatively high level of morbidity and need. Patients report an average of seven conditions, and nearly two thirds report general health that is either 'fair' or 'poor'. Patients were receiving a large number of medications and more than a third of participants reported anxiety or depression. Examining the baseline data also demonstrates that, consistent with previous literature, patients with multimorbidity are burdened by the demands placed on them by treatment and expectations of self-management.[39 40] Although there are many qualitative papers on the experience of patients with multimorbidity,[41] more quantitative data is needed. The trial recruitment procedures therefore identified a group of patients with significant burdens of illness and treatment whose characteristics seem well matched to the intervention model, and where many patients exceed minimum requirements of the trial eligibility criteria. Our data also suggest that patients do not receive care which they perceive as patient-centred in several important respects, as discussed below.

The third issue raised by this paper is an understanding of 'usual primary care' for multimorbidity in this population, to better understand current practice against which the potential benefits of 3D are being assessed. Assessing the 'nature of current care' for multimorbidity, and the degree to which it is 'patient centred care' is a complex task. Nevertheless, several important findings can be highlighted, linked to the 3D model (Figure 1).

Most patients reported satisfaction in general with their care. These high ratings are in line with wider work on patient perceptions of primary care and might indicate limited scope for improvement, but interpretation of such satisfaction scores is not always straightforward.[42] However, when considering the more structured aspects of care for long-term conditions (as assessed in models such as the Chronic Care Model[43]), the results showed more room for improvement. Many patients reported that their care was not always joined up and although three quarters of patients in this study had a preferred GP, only 59% reported that they usually consult them. The 3D model identifies eliciting and responding to the patient agenda (their own individual priorities) as a key gap in current care, and the questions from the LTC6 questionnaire and the PACIC scale showed only modest levels of agreement about items relating to this facet of care. This is in line with previous work in a broader population of patients.[44] Similarly, despite a very significant policy focus on care plans,[45] many practices did not have a policy to provide them and most patients did not report receiving them.

Many of the processes of care where we identified gaps (such as improving continuity and co-ordination of care, establishing the patient agenda to improve shared decision-making, production of care plans,) are a focus of the 3D model. If these processes are mediators of improvements in quality of life, as hypothesised by the logic model underlying the 3D approach, the trial may have a reasonable chance of seeing change in the intended primary and secondary outcomes, assuming it can be implemented.

Summary

The data suggest our pragmatic trial has achieved reasonable levels of external validity, and that the results should be generalizable to primary care in the United Kingdom. Although patients were generally satisfied with their relationships with primary care professionals, there remains significant room for improvement in important aspects of care for multimorbidity that are targeted by the 3D intervention. The pragmatic 3D randomised controlled trial will both test whether our intervention can generate enhancements in those processes of care, and whether those enhancements translate to better patient quality of life, patient experience and value for money.

Acknowledgements
The authors would like to thank Bristol Clinical Commissioning Group (CCG) for hosting this research, in particular Emma Moody, Joanne Atkinson, and Rebecca Robinson. We thank PRIMIS for developing the search. We would also like to thank members of the independent TSC, DMC, advisory group, and public and patient involvement group for their advice and input into the design and conduct of the study. Finally, we would like to thank the practices and patients and trainers for their participation.

Funding statement

This project was funded by the National Institute for Health Research Health Services and Delivery Research Programme (project number 12/130/15). This study was designed and conducted in collaboration with the Bristol Randomised Trials Collaboration (BRTC), a UKCRC Registered clinical trials unit (CTU) in receipt of National Institute for Health Research CTU support funding. CS is partly supported by The National Institute for Health Research Collaboration for Leadership in Applied Health Research and Care West.

For peer review only

Contributors

CS conceived the original study. CS, PB, SM, BG, IR, SB, AS and CM are co-applicants on the funding application. KC along with PB and CS led the writing of the first draft of the paper with contribution from MM (trial manager) and DG (statistical analyses). All authors contributed to the development of the editing of this manuscript.

Disclaimer

The views and opinions expressed therein are those of the authors and do not necessarily reflect those of the HS&DR Programme, NIHR, NHS or the Department of Health.

Competing interests None declared.
Ethics approval South-West (Frenchay) NHS Research Ethics Committee (14/SW/0011) and local NHS R&D approvals from the appropriate participating trusts. The views and opinions expressed therein

are those of the authors and do not necessarily reflect those of the HS&DR Programme, NIHR, NHS or the Department of Health.

Sponsor

The trial sponsor is the University of Bristol, (Senate House, Tyndall Avenue, Bristol BS8 1TH, UK)

Provenance and peer review Not commissioned; externally peer reviewed.

Data sharing statement Once the main results have been published, data may be available to other investigators subject to agreement about the protocol with the chief investigator and compliance with policies of the funder and sponsor in relation to data sharing.

For peer review only

References

1. National Guideline Centre. Multimorbidity: clinical assessment and management. London: National Institute for Health and Care Excellence, 2016.
2. Barnett K, Mercer S, Norbury M, et al. The epidemiology of multimorbidity in a large cross-sectional dataset: implications for health care, research and medical education. *Lancet* 2012;380:37-43.
3. Salisbury C, Johnson LR, Purdy S, et al. Epidemiology and impact of multimorbidity in primary care: a retrospective cohort study. *Br J Gen Pract* 2011;61(582):e12-e21. doi: 10.3399/bjgp11X548929
4. Palmer K, Marengoni A, Forjaz MJ, et al. Multimorbidity care model: Recommendations from the consensus meeting of the Joint Action on Chronic Diseases and Promoting Healthy Ageing across the Life Cycle (JA-CHRODIS). *Health Policy* 2018;122(1):4-11.
5. American Geriatric Society Expert Panel. Patient-centered care for older adults with multiple chronic conditions: a stepwise approach from the american geriatrics society: american geriatrics society expert panel on the care of older adults with multimorbidity. *J Am Geriatr Soc* 2012;60(10):1957-68. doi: 10.1111/j.1532-5415.2012.04187.x [published Online First: 2012/09/22]
6. Smith SM, Wallace E, O'Dowd T, et al. Interventions for improving outcomes in patients with multimorbidity in primary care and community settings. *Cochrane Database of Systematic Reviews* 2016(3) doi: 10.1002/14651858.CD006560.pub3
7. Muth C, van den Akker M, Blom JW, et al. The Ariadne principles: how to handle multimorbidity in primary care consultations. *BMC Medicine* 2014;12:223. doi: 10.1186/s12916-014-0223-1
8. Stokes J MM, Guthrie B, Mercer SW, Salisbury C & Bower P. The Foundations Framework for Developing and Reporting New Models of Care for Multimorbidity. *Annals of Family Medicine* 2017;15(6):570-77.
9. Salisbury C. Multimorbidity: time for action rather than words. *British Journal of General Practice* 2013;63(607):64-65. doi: 10.3399/bjgp13X661020
10. Naylor C, Parsonage M, McDaid D, et al. Long-term conditions and mental health: the cost of co-morbidities. London: The King's Fund, 2012.
11. Smith SM, Soubhi H, Fortin M, et al. Managing patients with multimorbidity: systematic review of interventions in primary care and community settings. *BMJ* 2012;345:e5205. doi: 10.1136/bmj.e5205 [published Online First: 2012/09/05]
12. Man MS, Chaplin K, Mann C, et al. Improving the management of multimorbidity in general practice: protocol of a cluster randomised controlled trial (The 3D Study). *Bmj Open* 2016;6(4):e011261. doi: 10.1136/bmjopen-2016-011261
13. Mann C SA, Guthrie B, Wye L, Man MS, Hollinghurst, S, Brookes S, Bower P, Mercer S & Salisbury C. Protocol for a process evaluation of a cluster randomised controlled trial to improve management of multimorbidity in general practice: the 3D study. *BMJ Open* 2016;6:e011260. doi: 10.1136/bmjopen-2016-011260
14. Stewart M. Towards a global definition of patient centred care. *BMJ* 2001;322(7284):444-45. doi: 10.1136/bmj.322.7284.444
15. Mead N, Bower P. Patient-centredness: a conceptual framework and review of the empirical literature. *Soc Sci Med* 2000;51(7):1087-110. [published Online First: 2000/09/27]
16. Craig P, Dieppe P, Macintyre S, et al. Developing and evaluating complex interventions: the new Medical Research Council guidance. *BMJ* 2008;337:a1655.
17. Loudon K, Treweek S, Sullivan F, et al. The PRECIS-2 tool: designing trials that are fit for purpose. *BMJ* 2015;350 doi: 10.1136/bmj.h2147
18. van Deudekom FJ, Postmus I, van der Ham DJ, et al. External validity of randomized controlled trials in older adults, a systematic review. *PloS one* 2017;12(3):e0174053.

For peer review only - http://bmjopen.bmj.com/site/about/guidelines.xhtml

19. Worsley SD, Rengerink KO, Irving E, et al. Series: Pragmatic trials and real world evidence: Paper 2. Setting, sites, and investigator selection. *Journal of Clinical Epidemiology* 2017(88):14-20.
20. Reeves D CS, Adams J, Shekelle PG, Kontopantelis E, Roland MO. Combining multiple indicators of clinical quality - An evaluation of different analytic approaches. *Med Care* 2007;45:489-96.
21. Zigmond AS, Snaith RP. The hospital anxiety and depression scale. *Acta Psychiatr Scand* 1983;67(6):361-70. [published Online First: 1983/06/01]
22. EuroQol Group. EuroQol Group EQ-5D-5L [Available from: http://www.euroqol.org/about-eq-5d/valuation-of-eq-5d/eq-5d-5l-value-sets.html accessed 11/02/2015.
23. Bayliss EA, Ellis JL, Steiner JF. Seniors' self-reported multimorbidity captured biopsychosocial factors not incorporated into two other data-based morbidity measures. *J Clin Epidemiol* 2009;62(5):550-57.e1. doi: 10.1016/j.jclinepi.2008.05.002
24. Duncan P MM, Man MS, Chaplin K, Gaunt D & Salisbury C. Development and validation of the Multimorbidity Treatment Burden Questionnaire (MTBQ). *BMJ Open*;In Press (submission ID bmjopen-2017-019413.R1)
25. Glasgow RE, Wagner EH, Schaefer J, et al. Development and validation of the Patient Assessment of Chronic Illness Care (PACIC). *Med Care* 2005;43(5):436-44. [published Online First: 2005/04/20]
26. Mercer SW, McConnachie A, Maxwell M, et al. Relevance and practical use of the Consultation and Relational Empathy (CARE) Measure in general practice. *Fam Pract* 2005;22(3):328-34. doi: 10.1093/fampra/cmh730
27. QIPP LTC Year of Care Funding Model Project Team. QIPP Long Term Conditions—Supporting the Local Implementation of the Year of Care Funding Model for People With Long-Term Conditions London, UK: Department of Health; 2012 [Available from: https://www.gov.uk/government/uploads/system/uploads/attachment_data/file/215060/dh_133652.pdf accessed 05/03/2018].
28. GP Patient Survey [Available from: www.gp-patient.co.uk accessed 05/03/2018].
29. NHS Digital. Quality and Outcomes Framework 2015 [Available from: http://content.digital.nhs.uk/catalogue/PUB18887 accessed 15/09/2017].
30. NHS Digital. Numbers of Patients Registered at a GP Practice 2015 [Available from: http://content.digital.nhs.uk/catalogue/PUB17356 accessed 05/07/2017].
31. ISD Scotland. GP workforce and practice list sizes 2015 [Available from: http://www.isdscotland.org/Health-Topics/General-Practice/Publications/data-tables.asp accessed 15/09/2017].
32. Public Health England. National General Practice Profiles 2015 [Available from: https://fingertips.phe.org.uk/profile/general-practice
33. Scottish Index of Mulitple Deprivation 2012 [Available from: http://geoconvert.mimas.ac.uk/ accessed 15/09/2017].
34. ISD Scotland. Quality and Outcomes Framework 2015 [Available from: https://www.isdscotland.org/Health-Topics/General-Practice/Quality-And-Outcomes-Framework/2014-15/practicelevel-summaries.asp accessed 15/09/2017].
35. NHS England. GP patient survey Practice Report 2016 [Available from: https://gp-patient.co.uk/practices-search accessed 14/03/2017].
36. NHS Scotland. Health and Care Experience Survey 2016 [Available from: http://www.hace15.quality-health.co.uk/index.php/reports/ accessed 15/09/2017].
37. Kennedy A, Bower P, Reeves D, et al. Implementation of self management support for long term conditions in routine primary care settings: cluster randomised controlled trial. *BMJ* 2013;346(f2882):1-11.
38. Coventry P, Lovell K, Dickens C, et al. Integrated primary care for patients with mental and physical multimorbidity: cluster randomised controlled trial of collaborative care for patients with depression comorbid with diabetes or cardiovascular disease. *BMJ* 2015;350:h638.

For peer review only

39. Gallacher KI, Batty GD, McLean G, et al. Stroke, multimorbidity and polypharmacy in a nationally representative sample of 1,424,378 patients in Scotland: implications for treatment burden. *BMC medicine* 2014;12(1):151.
40. May C, Montori VM, Mair FS. We need minimally disruptive medicine. *BMJ* 2009;339 doi: 10.1136/bmj.b2803
41. Rosbach M, Andersen JS. Patient-experienced burden of treatment in patients with multimorbidity–A systematic review of qualitative data. *PloS one* 2017;12(6):e0179916.
42. Burt J, Campbell J, Abel G, et al. Improving patient experience in primary care: a multimethod programme of research on the measurement and improvement of patient experience: NIHR Journals Library 2017.
43. Wagner EH, Austin BT, Von KM. Improving outcomes in chronic illness. *Manag Care Q* 1996;4(2):12-25.
44. Rick J, Rowe K, Hann M, et al. Psychometric properties of the patient assessment of chronic illness care measure: acceptability, reliability and validity in United Kingdom patients with long-term conditions. *BMC health services research* 2012;12(1):293.
45. Reeves D, Hann M, Rick J, et al. Care plans and care planning in the management of long-term conditions in the UK: a controlled prospective cohort study. *British Journal of General Practice* 2014;64(626):e568-e75.

For peer review only

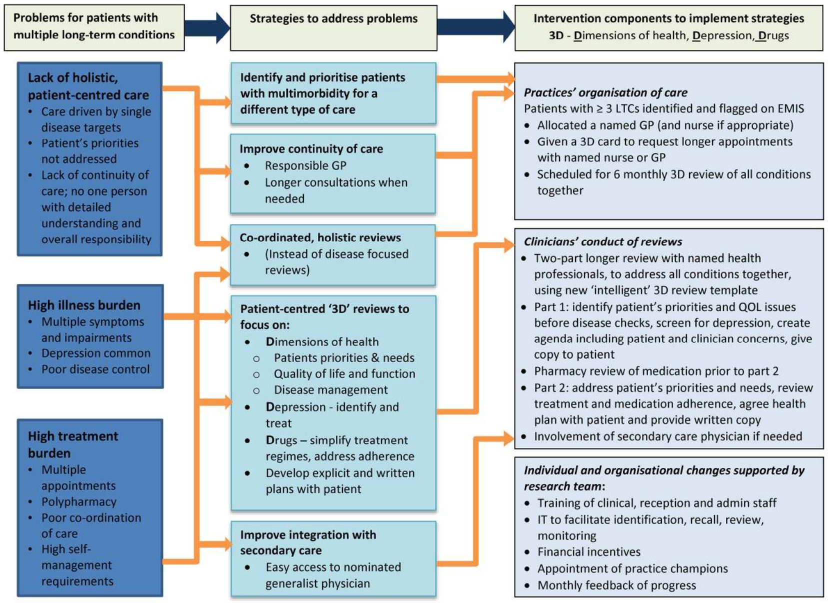

Figure 1: 3D logic model including theoretical mechanisms of action

209x148mm (300 x 300 DPI)

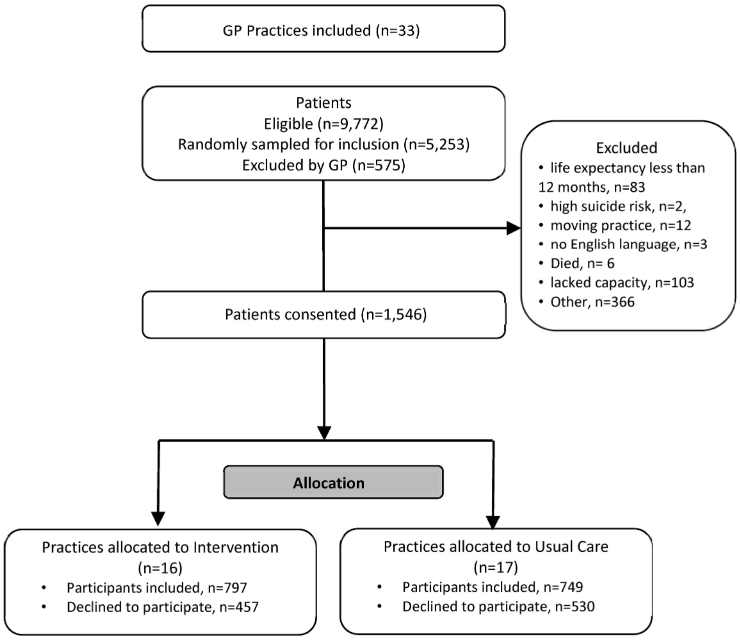

Figure 2: Flow of patients into the 3D trial

279x361mm (300 x 300 DPI)

Appendix A Chronic conditions for inclusion

We collected data on diagnoses of 17 conditions and combined these into 11 groups as shown below. Included patients must have diagnoses from three or more these groups of chronic conditions:

- Cardiovascular disease or Chronic kidney disease (including coronary heart disease, hypertension, heart failure, peripheral arterial disease, chronic kidney disease stage 3 to 5)*
- Stroke
- Diabetes
- Chronic Obstructive Pulmonary Disease or Asthma*
- Epilepsy
- Atrial fibrillation
- Severe mental health problems (schizophrenia or psychotic illness)*
- Depression
- Dementia
- Learning disability
- Rheumatoid arthritis

*Groups are counted only once even if a patient has multiple conditions within a group. For example, having both hypertension and heart failure would just count for one condition

For peer review only

Appendix B - 3D Study questionnaire

The 3D Study:
Improving whole person care

Practice: **Date:**

Please answer this questionnaire thinking about the patients with multi-morbidity in your practice. This will help us to understand your current approach and your perceptions about the care of these patients.

Name (optional): **Role (required):**

Please place an X in the box that is closest to your opinion	Strongly disagree	Disagree	Neither agree or disagree	Agree	Strongly agree
1. Patients' main concerns may be overlooked during review of their long-term conditions					
2. Depression is difficult to identify reliably without using a measure (such as PHQ9)					
3. Poly-pharmacy is difficult for patients to manage					
4. Multi-morbidity is difficult for clinicians to manage					
5. Patients with multi-morbidity have a special need for holistic, patient-centred care					
6. Holistic, patient-centred care is enhanced by continuity of care					
7. Patients with 3 or more conditions need longer appointments to address all their concerns					
8. Patients being reviewed for a long-term condition should be given a written care plan					
9. Patients prefer it if I make a plan, instead of asking them what they would like to do					
10. Patients are more likely to keep to goals and plans that they suggest themselves					
11. In this practice, the care patients receive for their long-term conditions is well-co-ordinated					
12. In this practice, review of long-term conditions is too disease-orientated and not holistic enough.					

For peer review only

Thank you for completing this questionnaire! Please return it to the 3D research team.

For peer review only - http://bmjopen.bmj.com/site/about/guidelines.xhtml

Table 1: CONSORT 2010 checklist of information to include when reporting a cluster randomised trial

Section/Topic	Item No	Standard Checklist item	Extension for cluster designs	Page No *
**Title and abstract**				
	1a	Identification as a randomised trial in the title	Identification as a cluster randomised trial in the title	1
	1b	Structured summary of trial design, methods, results, and conclusions (for specific guidance see CONSORT for abstracts)[1,2]	See table 2	2
**Introduction**				
**Background and objectives**	2a	Scientific background and explanation of rationale		5
			Rationale for using a cluster design	N/A
	2b	Specific objectives or hypotheses	Whether objectives pertain to the the cluster level, the individual participant level or both	6
**Methods**				
**Trial design**	3a	Description of trial design (such as parallel, factorial) including allocation ratio	Definition of cluster and description of how the design features apply to the clusters	7
	3b	Important changes to methods after trial commencement (such as eligibility criteria), with reasons		N/A
**Participants**	4a	Eligibility criteria for participants	Eligibility criteria for clusters	7
	4b	Settings and locations where the data were collected		7
**Interventions**	5	The interventions for each group with sufficient details to allow replication, including how and when	Whether interventions pertain to the cluster level, the individual participant level or both	N/A

		they were actually administered		
**Outcomes**	6a	Completely defined pre-specified primary and secondary outcome measures, including how and when they were assessed	Whether outcome measures pertain to the cluster level, the individual participant level or both	7-8
	6b	Any changes to trial outcomes after the trial commenced, with reasons		N/A
**Sample size**	7a	How sample size was determined	Method of calculation, number of clusters(s) (and whether equal or unequal cluster sizes are assumed), cluster size, a coefficient of intracluster correlation (ICC or *k*), and an indication of its uncertainty	N/A
	7b	When applicable, explanation of any interim analyses and stopping guidelines		N/A
**Randomisation:**				
**Sequence generation**	8a	Method used to generate the random allocation sequence		N/A
	8b	Type of randomisation; details of any restriction (such as blocking and block size)	Details of stratification or matching if used	N/A
**Allocation concealment mechanism**	9	Mechanism used to implement the random allocation sequence (such as sequentially numbered containers), describing any steps taken to conceal the sequence until interventions were assigned	Specification that allocation was based on clusters rather than individuals and whether allocation concealment (if any) was at the cluster level, the individual participant level or both	N/A
**Implementation**	10	Who generated the random allocation sequence, who enrolled participants, and who assigned participants to interventions	Replace by 10a, 10b and 10c	

	10a		Who generated the random allocation sequence, who enrolled clusters, and who assigned clusters to interventions	N/A
	10b		Mechanism by which individual participants were included in clusters for the purposes of the trial (such as complete enumeration, random sampling)	N/A
	10c		From whom consent was sought (representatives of the cluster, or individual cluster members, or both), and whether consent was sought before or after randomisation	7
**Blinding**	11a	If done, who was blinded after assignment to interventions (for example, participants, care providers, those assessing outcomes) and how		N/A
	11b	If relevant, description of the similarity of interventions		N/A
**Statistical methods**	12a	Statistical methods used to compare groups for primary and secondary outcomes	How clustering was taken into account	8
	12b	Methods for additional analyses, such as subgroup analyses and adjusted analyses		N/A
**Results**				
**Participant flow (a diagram is strongly recommended)**	13a	For each group, the numbers of participants who were randomly assigned, received intended treatment, and were analysed for the primary outcome	For each group, the numbers of clusters that were randomly assigned, received intended treatment, and were analysed for the primary outcome	Figure 2

For peer review only

	13b	For each group, losses and exclusions after randomisation, together with reasons	For each group, losses and exclusions for both clusters and individual cluster members	Figure 2
**Recruitment**	14a	Dates defining the periods of recruitment and follow-up		9
	14b	Why the trial ended or was stopped		N/A
**Baseline data**	15	A table showing baseline demographic and clinical characteristics for each group	Baseline characteristics for the individual and cluster levels as applicable for each group	Tables 2-6
**Numbers analysed**	16	For each group, number of participants (denominator) included in each analysis and whether the analysis was by original assigned groups	For each group, number of clusters included in each analysis	Tables 2-6
**Outcomes and estimation**	17a	For each primary and secondary outcome, results for each group, and the estimated effect size and its precision (such as 95% confidence interval)	Results at the individual or cluster level as applicable and a coefficient of intracluster correlation (ICC or k) for each primary outcome	Tables 2-6
	17b	For binary outcomes, presentation of both absolute and relative effect sizes is recommended		
**Ancillary analyses**	18	Results of any other analyses performed, including subgroup analyses and adjusted analyses, distinguishing pre-specified from exploratory		N/A
**Harms**	19	All important harms or unintended effects in each group (for specific guidance see CONSORT for harms[3])		N/A
**Discussion**				
**Limitations**	20	Trial limitations, addressing sources of potential bias,		12

		imprecision, and, if relevant, multiplicity of analyses		
**Generalisability**	21	Generalisability (external validity, applicability) of the trial findings	Generalisability to clusters and/or individual participants (as relevant)	12
**Interpretation**	22	Interpretation consistent with results, balancing benefits and harms, and considering other relevant evidence		12-14
**Other information**				
**Registration**	23	Registration number and name of trial registry		2
**Protocol**	24	Where the full trial protocol can be accessed, if available		Supplement 2
**Funding**	25	Sources of funding and other support (such as supply of drugs), role of funders		14

** Note: page numbers optional depending on journal requirements*

For peer review only

Table 2: Extension of CONSORT for abstracts[1,2] to reports of cluster randomised trials

Item	Standard Checklist item	Extension for cluster trials
**Title**	Identification of study as randomised	**Identification of study as cluster randomised**
**Trial design**	Description of the trial design (e.g. parallel, cluster, non-inferiority)	
**Methods**		
**Participants**	Eligibility criteria for participants and the settings where the data were collected	**Eligibility criteria for clusters**
**Interventions**	Interventions intended for each group	
**Objective**	Specific objective or hypothesis	**Whether objective or hypothesis pertains to the cluster level, the individual participant level or both**
**Outcome**	Clearly defined primary outcome for this report	**Whether the primary outcome pertains to the cluster level, the individual participant level or both**
**Randomization**	How participants were allocated to interventions	**How clusters were allocated to interventions**
**Blinding (masking)**	Whether or not participants, care givers, and those assessing the outcomes were blinded to group assignment	
**Results**		
**Numbers randomized**	Number of participants randomized to each group	**Number of clusters randomized to each group**
**Recruitment**	Trial status[1]	
**Numbers analysed**	Number of participants analysed in each group	**Number of clusters analysed in each group**
**Outcome**	For the primary outcome, a result for each group and the estimated effect size and its precision	**Results at the cluster or individual participant level as applicable for each primary outcome**
**Harms**	Important adverse events or side effects	
**Conclusions**	General interpretation of the results	
**Trial registration**	Registration number and name of trial register	
**Funding**	Source of funding	

[1] Relevant to Conference Abstracts

REFERENCES

1 Hopewell S, Clarke M, Moher D, Wager E, Middleton P, Altman DG, et al. CONSORT for reporting randomised trials in journal and conference abstracts. *Lancet* 2008, 371:281-283

2 Hopewell S, Clarke M, Moher D, Wager E, Middleton P, Altman DG at al (2008) CONSORT for reporting randomized controlled trials in journal and conference abstracts: explanation and elaboration. *PLoS Med* 5(1): e20

3 Ioannidis JP, Evans SJ, Gotzsche PC, O'Neill RT, Altman DG, Schulz K, Moher D. Better reporting of harms in randomized trials: an extension of the CONSORT statement. *Ann Intern Med* 2004; 141(10):781-788.

For peer review only
